# Polysulfides made from re-purposed waste are sustainable materials for removing iron from water†

**DOI:** 10.1039/c7ra11999b

**Published:** 2018-01-03

**Authors:** Nicholas A. Lundquist, Max J. H. Worthington, Nick Adamson, Christopher T. Gibson, Martin R. Johnston, Amanda V. Ellis, Justin M. Chalker

**Affiliations:** Centre for NanoScale Science and Technology, College of Science and Engineering, Flinders University Sturt Road Bedford Park South Australia 5042 Australia justin.chalker@flinders.edu.au; School of Chemical and Biomedical Engineering, University of Melbourne Parkville Victoria 3010 Australia

## Abstract

Water contaminated with Fe^3+^ is undesirable because it can result in discoloured plumbing fixtures, clogging, and a poor taste and aesthetic profile for drinking water. At high levels, Fe^3+^ can also promote the growth of unwanted bacteria, so environmental agencies and water authorities typically regulate the amount of Fe^3+^ in municipal water and wastewater. Here, polysulfide sorbents—prepared from elemental sulfur and unsaturated cooking oils—are used to remove Fe^3+^ contaminants from water. The sorbent is low-cost and sustainable, as it can be prepared entirely from waste. The preparation of this material using microwave heating and its application in iron capture are two important advances in the growing field of sulfur polymers.

## Abstract 2

A polymer prepared by co-polymerisation of sulfur and canola oil removed Fe^3+^ from water. Microwave irradiation was convenient in promoting the polymerisation.

## Graphical abstract

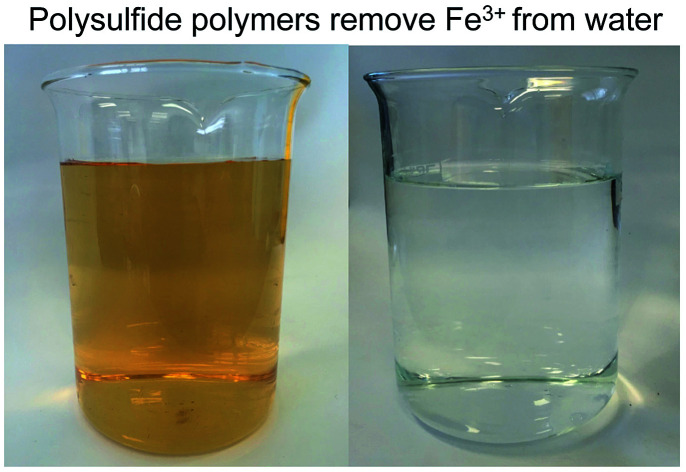

The removal of iron from groundwater, potable water and wastewater is an important requirement for water authorities, industry, and environmental agencies.^1^ Ferric iron (Fe^3+^) in particular is undesirable because it leads to discolouration of plumbing fixtures^2,3^ and imparts unappealing odour and taste to drinking water.^2^ Additionally, Fe^3+^ promotes the growth of certain bacteria, leading to fouling, clogging of pipes and other undesirable ecological effects.^4^ There are also some indications that adverse health effects in aquatic life may originate from high levels of iron.^5,6^ The control of iron content in water is therefore an important economic and environmental issue, with levels typically regulated by government authorities.^7,8^ There are several methods for removing iron from water including ion exchange,^9^ oxidative precipitation with subsequent flocculation and/or filtration,^10,11^ and adsorption of iron onto activated carbon,^12^ among other techniques.^1^ However, in scenarios where large volumes of water are treated, these methods may be expensive, low in performance, or both.^1^

Our interest in controlling iron content in water stemmed from a recent engagement with an industry partner facing a challenge in meeting regulatory requirements for iron levels in groundwater pumped and discharged from an underground operations centre. A combination of oxidative conversion of Fe^2+^ to Fe^3+^ and separation using flocculants and filters provided some success in meeting the 3 mg L^−1^ daily discharge limit, but alternative low-cost methods were desired to facilitate the treatment and discharge of more than 150 000 L per day containing iron levels in the range of 35–60 mg L^−1^. Our laboratory has recently reported the use of inexpensive polymers made from elemental sulfur and their use in sequestering metals such as palladium and mercury.^13–15^ It was therefore intriguing scientifically, economically and environmentally to determine if these polymers were suitable in the removal of iron from water.

Sulfur polymers, especially those prepared by inverse vulcanisation and related processes,^16^ have emerged as versatile materials in diverse areas of science.^15–20^ These studies are motivated, at least in part, by the megaton stores of sulfur available from crude oil desulfurisation.^21,22^ In converting this petroleum by-product into useful polymers, interesting applications have since been reported in power generation^23,24^ and storage,^16^ high refractive index and IR transmitting optical devices,^25–27^ dynamic and healable materials,^26,28^ thermal insulation,^29^ sulfur-doped carbon materials,^30^ and heavy metal remediation.^13,14,29–33^

In the case of metal binding, polysulfide polymers have been used primarily to sequester highly toxic mercury salts.^13,14,31–33^ Because these sulfur polymers are simple to prepare in a single chemical step, we hypothesised that even if the affinity of the polysulfide for the harder Fe^3+^ is lower than for the softer Hg^2+^, the polymer may still be useful in removing the former metal from water. This hypothesis is further motivated by Theato's recent discovery that while high-sulfur polymers are excellent at capturing mercury, there is still appreciable binding to Fe^3+^ for an electrospun blend of a poly(sulfur-statistical-diisopropenylbenzene) polysulfide and poly(methyl methacrylate).^32^

We therefore set out to assess the iron-binding properties of a different co-polymer prepared by the direct reaction of elemental sulfur and canola oil. Importantly, this co-polymer is sustainably synthesised using only sulfur and the widely available and renewable canola oil.^34–36^ The resulting material is an elastomeric, high-sulfur factice^37^ that has previously been studied by Theato as a novel cathode material^38^ and by our team as a reactive sorbent for mercury pollution.^14^ Should this material prove effective in removing Fe^3+^ from water, it would represent a cost-effective method for water treatment through waste valorisation.

As a starting point, the canola oil polysulfide was synthesised by first heating sulfur to 180 °C in order to promote radical ring-opening polymerisation.^14^ Canola oil was then added to the polysulfide pre-polymer to cross-link the sulfur chains. The reaction mixture typically reaches its gel point within 20 minutes, at which time the reaction is cooled to provide a rubber-like material. The synthesis was prepared for 50%, 60%, and 70% sulfur by mass. The polymer was then milled into 1.0–2.5 mm diameter particles for subsequent use (Fig. 1 and S4†). Analysis of the polymers by infrared spectroscopy and Raman spectroscopy were consistent with our previous report on the material, in which the key polysulfide structure (S–S bonds) and canola oil backbone groups (*e.g.* C=O) are evident (S5–S6†).^14^ Thermogravimetric analysis and differential scanning calorimetry were also consistent with that previously reported for these materials, with major decompositions initiated over 200 °C. The first of these decompositions corresponds to thermal breakdown of S–S bonds and the second major mass loss above 300 °C corresponds to the decomposition of the remaining canola oil domain of the polymer (S7†). SEM analysis of the polysulfides indicated a smooth polymer surface embedded with high-sulfur micron scale particles (S8–S10†).^14,38^

**Fig. 1:**
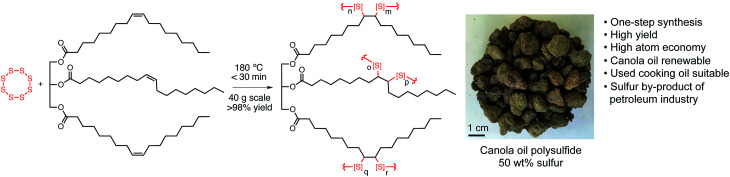
A canola oil polysulfide was prepared by direct reaction of canola oil and elemental sulfur. The material was prepared with 50, 60, and 70% sulfur by mass for subsequent iron sorption studies (50% sulfur polymer shown). An approximate structure of canola oil is shown, with oleic acid as the major fatty acid in the triglyceride.

With the polymer in hand, we then tested its ability to bind to and remove Fe^3+^ from water. An aqueous solution of FeCl_3_ was prepared at a concentration of 50 mg L^−1^ and then equilibrated for 48 hours. The resulting solution (pH 3.0) could be monitored by its absorbance at 306 nm. At this composition, the Fe^3+^ is fully soluble so that any reduction in iron content over time could be attributed to binding to the polymer, rather than precipitation. Accordingly, 2.0 g of the polysulfide was added to a 20 mL sample of the Fe^3+^ solution to benchmark iron removal efficiency. After 24 hours of incubation with gentle agitation (end over end mixing), the absorbance of the solution at 310 nm was measured to determine the amount of Fe^3+^ captured by the polymer. The concentration of the iron was typically reduced to between 3 and 6 mg L^−1^ for all polymer samples (50, 60 and 70% sulfur, S11–S12†). Subsequent experiments were therefore restricted to the polysulfide prepared at 50% sulfur, 50% canola oil by mass. At this composition, the particles are more elastic and durable; at higher levels of sulfur the particles become more brittle.

The amount of polymer in these initial tests (2.0 g per 20 mL of water) was somewhat arbitrary, but subsequent testing revealed that this mass was indeed required to reduce the iron concentration below 10 mg L^−1^ (S13–S14†). SEM and XRD analysis of the polymer after the water treatment revealed no morphological change (S15–S16†), indicating stability of the polymer structure during the treatment. It should also be noted that relatively low levels of iron are actually bound to the polymer (less than 1 mg Fe^3+^ per 2 g of polymer in these experiments). Nevertheless, the ease at which this polymer can be prepared on relatively large scales allowed a demonstration of a 1 L scale water purification in which the iron concentration was reduced from 50 mg L^−1^ to 1.3 mg L^−1^, as measured independently by UV-vis analysis and atomic absorption spectroscopy (Fig. 2). Furthermore, exposing the polymer to hydrogen peroxide did not reduce its ability to remove Fe^3+^ from water—an important feature that makes the material compatible with many iron removal process that rely on the conversion of Fe^2+^ to Fe^3+^ through reaction with hydrogen peroxide (S17–S18†). This demonstration is important because the polymer was not effective in removing Fe^2+^ from water, with no significant reduction in Fe^2+^, as indicated by AAS (S21†). Another beneficial feature of the polymer (in comparison to elemental sulfur) is that the polymer particles are not prone to caking, which makes filtration a straightforward process (S20†).

**Fig. 2:**
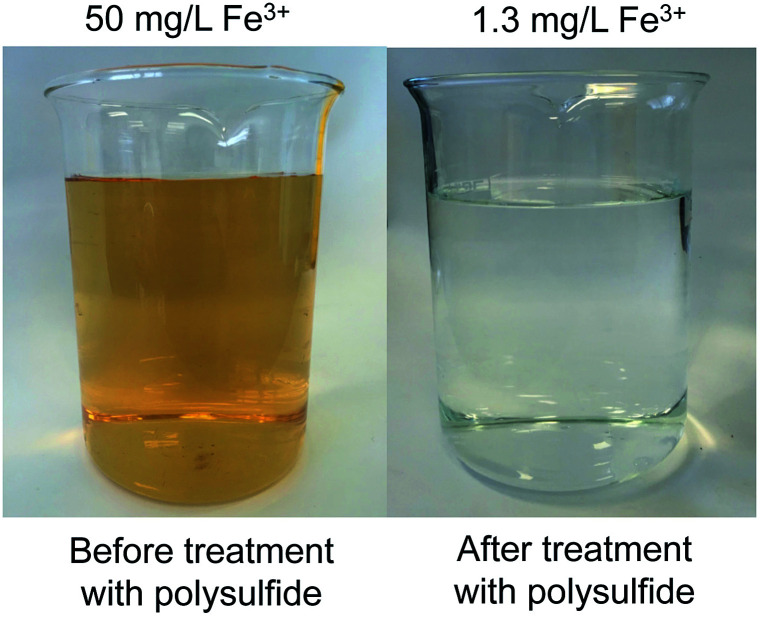
A 1.0 L solution of Fe^3+^ (50 mg L^−1^) was treated with 200 g of the non-porous canola oil polysulfide (50% sulfur) for 24 hours at 23 °C. The polymer reduced the iron concentration to 1.3 mg L^−1^, as measured independently by UV-vis spectroscopy and atomic absorption spectroscopy (AAS). After removing the polysulfide by filtration, the treated water appears colourless. See S19† for additional details.

Because relatively large amounts of the polysulfide are required to remove Fe^3+^ from water, we anticipated that increasing surface area would improve its performance. Accordingly, a porous version of the polysulfide was prepared by reacting sulfur and canola oil in the presence of a large excess of a sodium chloride porogen (70% of the reaction mixture is sodium chloride). Soaking the resulting product in water removes the porogen, leaving micron scale pores and channels in the polysulfide material (Fig. 3a and S22†). This material was consistent in its thermal and spectroscopic properties to those previously reported when it was prepared for mercury sorption (S22–S24†).^14^ This material was also stable across a wide pH range at 25 °C, with minimal decomposition after incubation for 7 days in water at pH 1, 6 or 13, as indicated by visual inspection and ^1^H NMR analysis (S25–S27†).

**Fig. 3:**
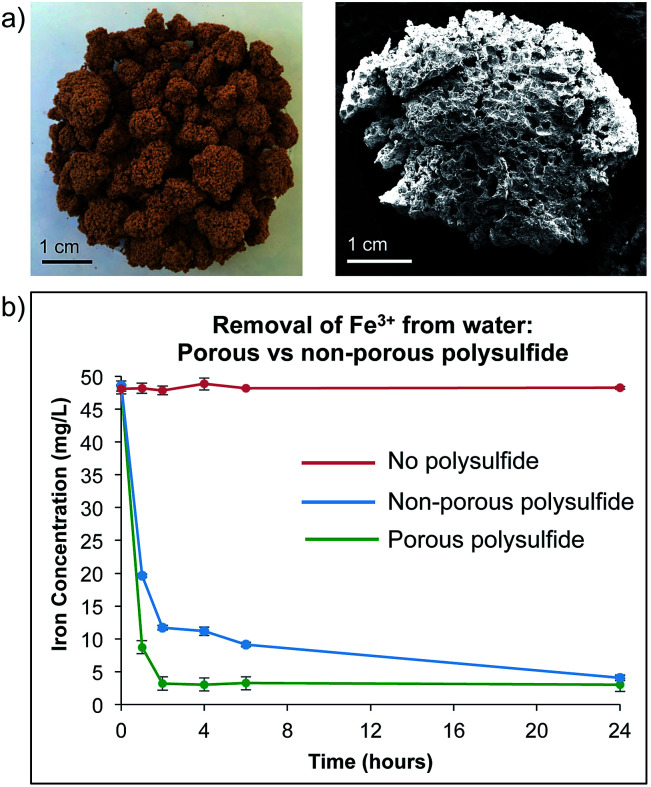
A porous version of the canola oil polysulfide was prepared by reacting canola oil and sulfur in the presence of a sodium chloride porogen. Sodium chloride was removed from the polymer by washing with water, resulting in a polymer with micron-scale pores. (a) Photograph and SEM micrograph of the porous canola oil polysulfide. (b) Fe^3+^ sorption over time for the porous polysulfide (green plot) and non-porous polysulfide (blue plot). The iron concentration did not change if no polymer was added to the solution (red plot).

With the porous canola oil polysulfide in hand, the Fe^3+^ capture experiments were repeated and compared to the non-porous polymer. The porous polymer was superior in the rate of Fe^3+^ removal from water and less polymer was required. For example, 2.0 g of the porous polysulfide was able to reduce the Fe^3+^ concentration in a 20 mL sample from 50 mg L^−1^ to 3 mg L^−1^ in 2 hours (Fig. 3b). It was also demonstrated that only 1.0 g of the polymer was required to reduce the concentration of Fe^3+^ from 50 mg L^−1^ to 3 mg L^−1^ for 20 mL of contaminated water (S28–S30†). Fitting the sorption data to a Langmuir isotherm model indicated a sorption capacity of 0.8 mg g^−1^ (S31†). The reduction of iron concentration was also demonstrated across a pH range of 1 to 10, though precipitation also accounts for the reduction in iron concentration above pH 3.0 (S32†). Even in these cases, the polysulfide is beneficial and serves as a filtration media to prevent caking when removing precipitated iron salts by filtration. Importantly, the polysulfide's ability to remove iron from water was not impacted by the presence of other common ions such as Na^+^, K^+^, Mg^2+^, Ca^2+^, and Cl^−^, even when all were present at 10 mg L^−1^ in the Fe^3+^ solution (S33–S34†).

If the polymer was re-used in the same iron sorption experiment, the sorption capacity dropped significantly with 95% Fe^3+^ removal on the first run and 62% and 26% on the second and third uses, respectively (S36†). This result suggests that the polymer is best deployed as a single use material in Fe^3+^ binding. Fortunately, when the polymer was prepared from waste cooking oil and sulfur (S37–S39†), it was equally as effective in removing Fe^3+^ from water (S40†). This result means that even though the polymer is best used for a single water treatment, this is a productive example of waste valorisation. We are currently investigating how the polymer–iron complex can be re-purposed yet again as an additive in construction materials and novel composites.

To deploy sulfur polymers for applications in water purification and environmental chemistry, it is useful to have methods to prepare the material rapidly and on-demand. Because sulfur-based polymers are excellent thermal insulators,^29^ it is difficult to ensure even and reliable heating during the polymerisation. Addressing this issue, we investigated whether microwave irradiation would be practical in the synthesis of the canola oil polysulfide. Because canola oil and unsaturated triglycerides can be heated rapidly with microwave irradiation, we hypothesised that this might be a convenient strategy for heating the reaction mixture. Indeed, both laboratory microwave reactors with precise temperature and power control (S41–S42†) and conventional household microwave ovens (Fig. 4 and S43†) were highly effective for the rapid heating and subsequent co-polymerisation of canola oil and sulfur. For instance, when the polymerisation was carried out in an 1100 W household microwave oven, the polymer was formed within a mere 5 minutes. The spectroscopic, thermal and Fe^3+^ binding properties of the material synthesised in the microwave reactor were no different from the material prepared through the slower conventional heating (S43–S47†). The ability to prepare these polymers in a conventional microwave may be important in scaling up the synthesis of these polymers because several batches could be prepared in rapid succession or through the use of several inexpensive microwave reactors operating in parallel. This process might also make the polysulfide accessible in areas with limited resources—an important consideration when the polymers will be used to remove heavy metals from water.^14^

**Fig. 4:**
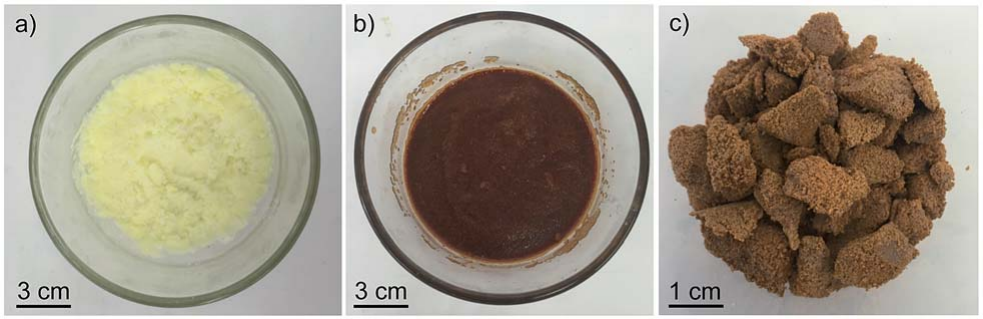
(a) Reaction mixture to form a porous polysulfide: canola oil (15.0 g), sulfur (15.0 g), and sodium chloride (70.0 g). (b) Product of polymerisation after irradiation in a household microwave (1100 W) for 5 minutes. (c) Porous polysulfide obtained after removing the sodium chloride porogen with a water wash.

In summary, a polysulfide material made from canola oil and sulfur was used to remove Fe^3+^ from water. The iron removal was tested at industrially relevant concentrations and purified to levels within the limits of environmental regulatory agencies. A rapid and scalable synthesis of the polysulfide was also executed in a microwave reactor—an important milestone in the synthesis of high-sulfur polymers because of the challenge in reliably and evenly heating these thermally insulating materials. More generally, because the featured polymer can be made entirely from industrial waste, this study is an advance in sustainable chemistry, waste valorisation, and environmental chemistry.

## Conflicts of interest

Two authors (M. J. H. W. and J. M. C.) are inventors on a patent associated with the synthesis and applications of the canola oil polysulfide material (Patent No. WO 2017181217).

## Supplementary Material

RA-008-C7RA11999B-s001
