# Supplementary material for: Polysulfides made from re-purposed waste are sustainable materials for removing iron from water
Source: RSC Adv. 2018 Jan 3;8(3):1232–6. doi: 10.1039/c7ra11999b (PMC9077003; doi:10.1039/c7ra11999b)
Supplement: RA-008-C7RA11999B-s001 [file RA-008-C7RA11999B-s001.pdf]

Electronic Supplementary Material (ESI) for RSC Advances.
This journal is © The Royal Society of Chemistry 2017

Polysulfides made from re-purposed waste are sustainable materials for removing iron from water

Nicholas A. Lundquist,[a] Max J. H. Worthington,[a] Nick Adamson,[a,b] Christopher T. Gibson,[a] Martin R. Johnston,[a] Amanda V. Ellis[a,b] and Justin M. Chalker*[a,b]

a. Centre for NanoScale Science and Technology, College of Science and Engineering, Flinders University, Sturt Road, Bedford Park, South Australia, 5042, Australia. E-mail: justin.chalker@flinders.edu.au

b. School of Chemical and Biomedical Engineering, University of Melbourne, Parkville, Victoria, 3010, Australia

Table of Contents

General considerations S2
Canola oil polysulfide synthesis (non-porous) S3
IR spectroscopic analysis (non-porous polysulfide) S5
Raman spectroscopic analysis (non-porous polysulfide) S5
Differential Scanning Calorimetry (DSC) for non-porous canola oil polysulfides S7
Thermogravimetric Analysis (TGA) for non-porous canola oil polysulfides S7
SEM analysis of non-porous canola oil polysulfides S8
Fe(III) calibration curve for UV-Vis analysis S11
Fe(III) removal from water using non-porous polysulfides of varying sulfur content S12
Fe(III) removal from water using varying mass of 50% sulfur non-porous polysulfide S13
Optimised Fe(III) removal process using non-porous polysulfide (50 wt% sulfur) S14
SEM and XRD analysis of non-porous polymer after Fe(III) removal process S15
Treatment of non-porous polysulfide with hydrogen peroxide S17
Removal of Fe(III) from water using non-porous polysulfide previously treated with H_2O_2 S18
Litre-scale Fe(III) removal from water using the non-porous polysulfide (50 wt% sulfur) S19
Comparison of elemental sulfur and the non-porous polysulfide mixing in water S20
Fe(II) removal from water using the non-porous polysulfide (50 wt% sulfur) S21
Porous canola oil polysulfide synthesis (50 wt% sulfur) S22
SEM analysis of porous canola oil polysulfide (50 wt% sulfur) S23
IR analysis of porous canola oil polysulfide (50 wt% sulfur) S23
XRD analysis of porous canola oil polysulfide (50 wt% sulfur) S24
Stability of porous canola oil polysulfide at different pH S25
Fe(III) removal from water using the porous canola oil polysulfide (50 wt% sulfur) S28
Fe(III) removal from water: porous vs non-porous polysulfide S29
Fe(III) removal from water: varying mass of porous polysulfide (50 wt% sulfur) S30
Langmuir sorption isotherm for porous polysulfide (50 wt% sulfur) S31
Effect of pH on the efficiency of Fe(III) removal using the porous polysulfide (50 wt% sulfur) S32
The effect of 'contaminant' ions on the removal of Fe(III) from water S33
Kinetic analysis of Fe(III) sorption onto the porous polysulfide (50 wt% sulfur) S34
Re-use of the porous polysulfide (50 wt% sulfur) in Fe(III) removal from water S36
Synthesis and characterisation of a porous polysulfide using recovered cooking oil S37
Fe(III) removal from water using a porous polysulfide prepared from recovered cooking oil S40
Synthesis and characterisation of a porous polysulfide using a microwave reactor S41
Synthesis and characterisation of a porous polysulfide using a household microwave S43
IR analysis of porous polysulfide (50 wt% sulfur) prepared with microwave irradiation S44
SEM analysis of porous polysulfide (50 wt% sulfur) prepared with microwave irradiation S45
STA analysis of porous polysulfide (50 wt% sulfur) prepared with microwave irradiation S46
Fe(III) treatment using a polysulfide prepared using microwave irradiation S47
References S47

General considerations

IR Spectroscopy: Infrared (IR) spectra were recorded on a Fourier Transform spectrophotometer using the ATR method. Transmission maxima (υ_{max}) are reported in wavenumbers (cm^{-1}).

Raman Spectroscopy: Raman spectra were acquired using a Witec alpha300R Raman microscope at an excitation laser wavelength of 532 nm with a 40X objective (numerical aperture 0.60). Typical integration times for single Raman spectra were between 20 to 60 s and averaged from 1 to 3 repetitions.

SEM and EDS: Scanning Electron Microscopy (SEM) images were obtained using an FEI F50 Inspect system, while corresponding EDS spectra were obtained using an EDAX Octane Pro detector.

Differential Scanning Calorimetry (DSC): Differential Scanning Calorimetry (DSC) was carried out using a Perkin Elmer DSC 8000 with nitrogen furnace purged at 20 mL/min. Samples were approximately 7 mg and sealed in aluminium sample pans. The sample was cooled to -80 ºC, held for 5 minutes, and then heated to 300 ºC at 10 ºC/min.

Thermogravimetric Analysis (TGA): Simultaneous Thermal Analysis (STA) was carried out on a Perkin Elmer STA8000 simultaneous thermal analyzer (STA). A sample size between 11 and 15 mg was used in each run. The furnace was purged at 20 mL/min with nitrogen, and equilibrated for 1 minute at 30 ºC before each run. Heating was carried out up to 700 ºC using a 20 ºC/min heating rate. The temperature was held isothermally at 700 ºC at the end of each experiment to oxidize remaining organic matter.

X-ray diffraction: Powder X-ray diffraction (XRD) patterns were recorded on a Bruker D8 Advance Eco diffractometer (Bragg-Brentano geometry) using Co-Kα radiation (λ = 1.78897 Å). The Bragg angle (2θ) was varied from 15° to 90° with a step size of 0.019°, measurement time of 0.45 s per step and sample rotation at 15 rpm. The XRD patterns were collected on a silicon low background sample holder, where powder samples were deposited onto the surface of the holder.

UV-Vis spectroscopy:
UV-Vis spectroscopy was performed using an Agilent Technologies Cary 60 UV-vis spectrometer. A sample size of 1 ml was used for each measurement. All measurements were initially referenced to pure water before sample measurement. Fe(III) was monitored at 306 nm.

Atomic Absorption Spectroscopy (AAS):
Atomic absorption spectroscopy was performed using a GBC 933 plus atomic absorptions spectrometer with a Fe hollow cathode lamp. Analysis of iron concentrations between 2-9 mg/L was performed at a wavelength of 248.3 nm. Analysis of iron concentrations between 20-80 mg/L was performed using a wavelength of 372 nm. A sample size of approximately 3-5 ml was required for each measurement. Every sample was first referenced to pure water before sample analysis.

Canola oil polysulfide synthesis (non-porous)

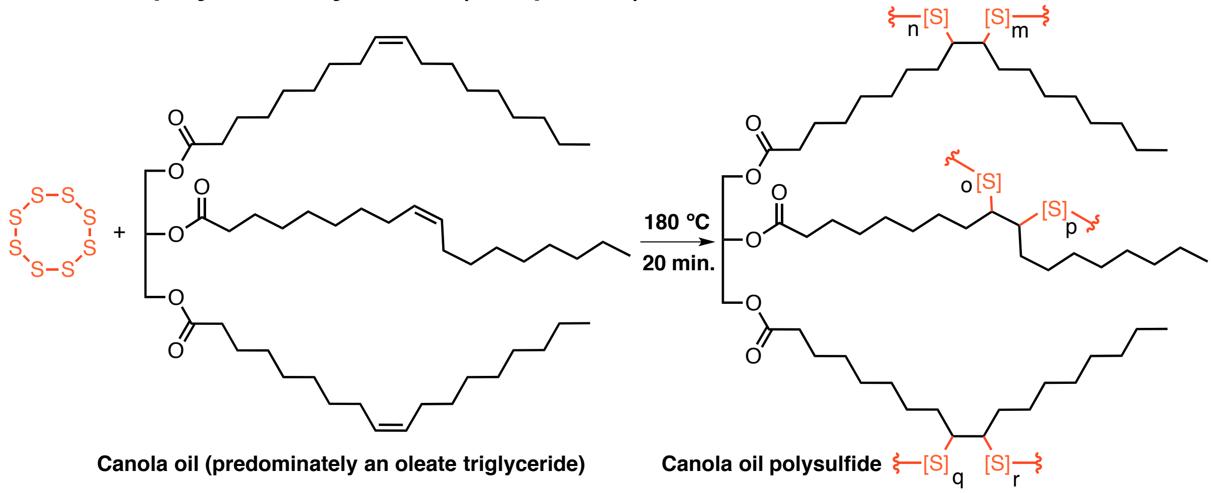

Simplified structure of the canola oil and the canola oil polysulfide. Note that the polysulfide may potentially crosslink both inter- and intramolecularly.

The polysulfide synthesis was adapted from our previously published procedure.[1]
Polysulfide (50% sulfur): Sulfur (technical grade, 20.0 g) was added to a 250 mL round bottom flask and then melted, with stirring, before heating further to 180 °C. Canola oil (20.0 g) was then added dropwise over 3-5 minutes, resulting in a two-phase mixture. The reaction was stirred vigorously to ensure efficient mixing of the two phases. The mixture appeared to form one phase after approximately 10 minutes. Heating was continued for an additional 10 minutes at 180 °C. Over this time, the product formed a rubbery solid. The material was then removed from the flask and then blended for 2-3 minutes (8.5 cm rotating blade) to provide rubber particles less than 12 mm in diameter. The particles were then transferred to a beaker and treated with enough 0.1 M NaOH to cover the particles entirely. This mixture was stirred for 90 minutes at room temperature to remove any residual hydrogen sulfide. Care was taken to submerge any polymer particles that floated on the NaOH solution. After this time, the particles were isolated by filtration and then washed on the filter with deionised water (3 × ~50 mL). The particles were then collected from the filter and air dried at room temperature and pressure for 24 hours. Typically, this procedure provided a final mass between 39.2 and 40.0 g of the washed and dried canola oil polysulfide particles (>98% yield). The polymer particles were then partitioned into various size distributions using sieves. The particle size ranges obtained were < 0.5 mm, 0.5-1.0 mm, 1.0-2.5 mm, and 2.5-5.0 mm, and > 5.0 mm. For all subsequent experiments, the particles ranging from 1.0-2.5 mm were used. This size was selected to balance the need for higher surface area (smaller particles), but also to minimise caking and hydraulic resistance associated with smaller particles. A similar protocol was used for the synthesis of the 60% and 70% sulfur polymers. For the 60% sulfur polymer, 16.0 g canola oil and 24.0 g of sulfur were used. For the 70% sulfur polymer, 12.0 g canola oil and 28.0 g of sulfur were used.

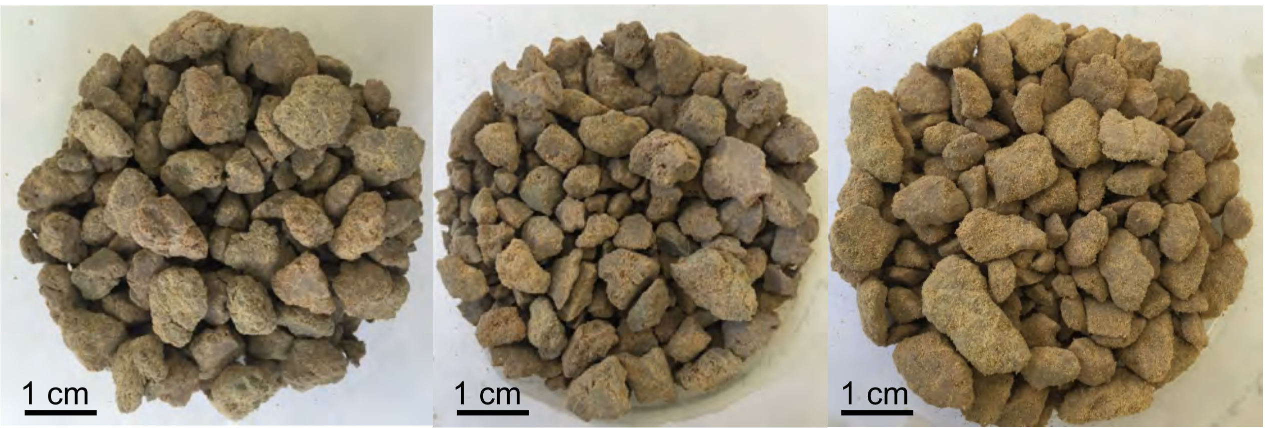

Figure S1: Images of non-porous polysulfide particles (left to right: 50, 60, and 70 wt% sulfur)

Figure S2: IR Spectra for non-porous canola oil polysulfides

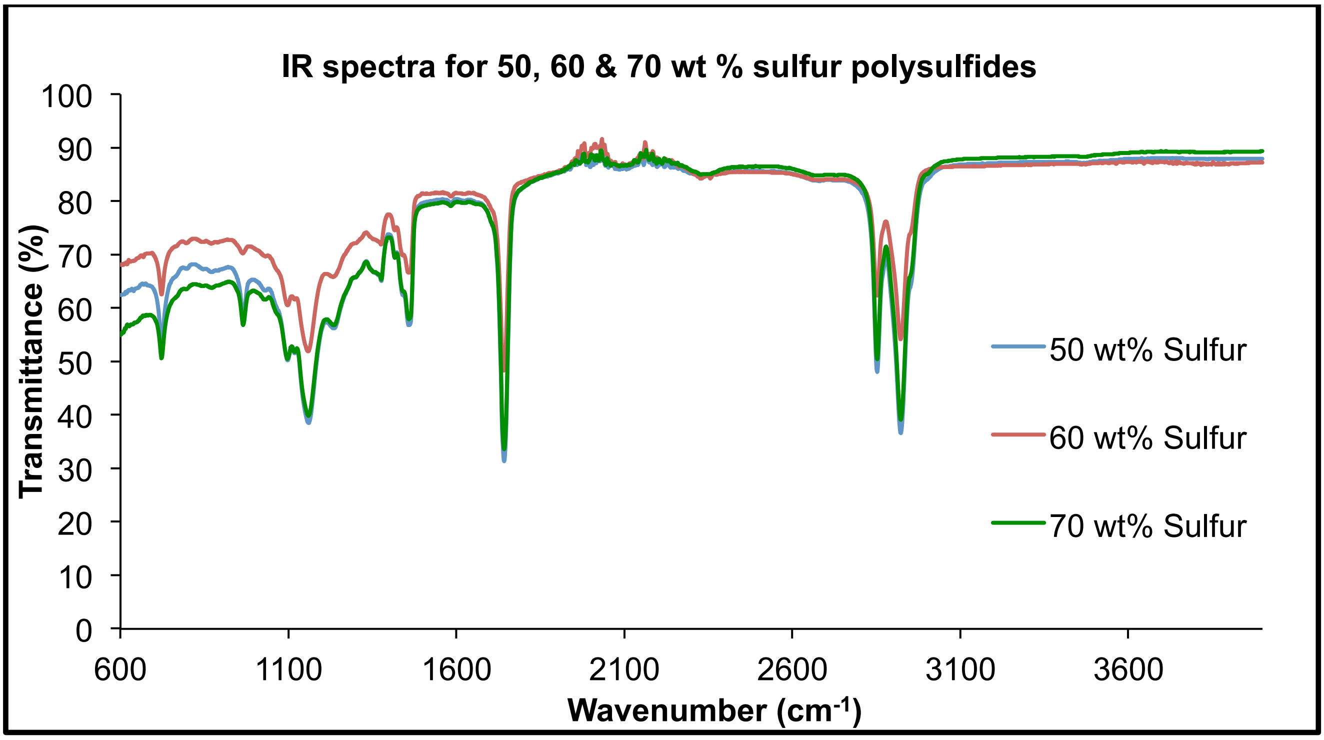

Figure S3: Raman Spectra for non-porous canola oil polysulfides

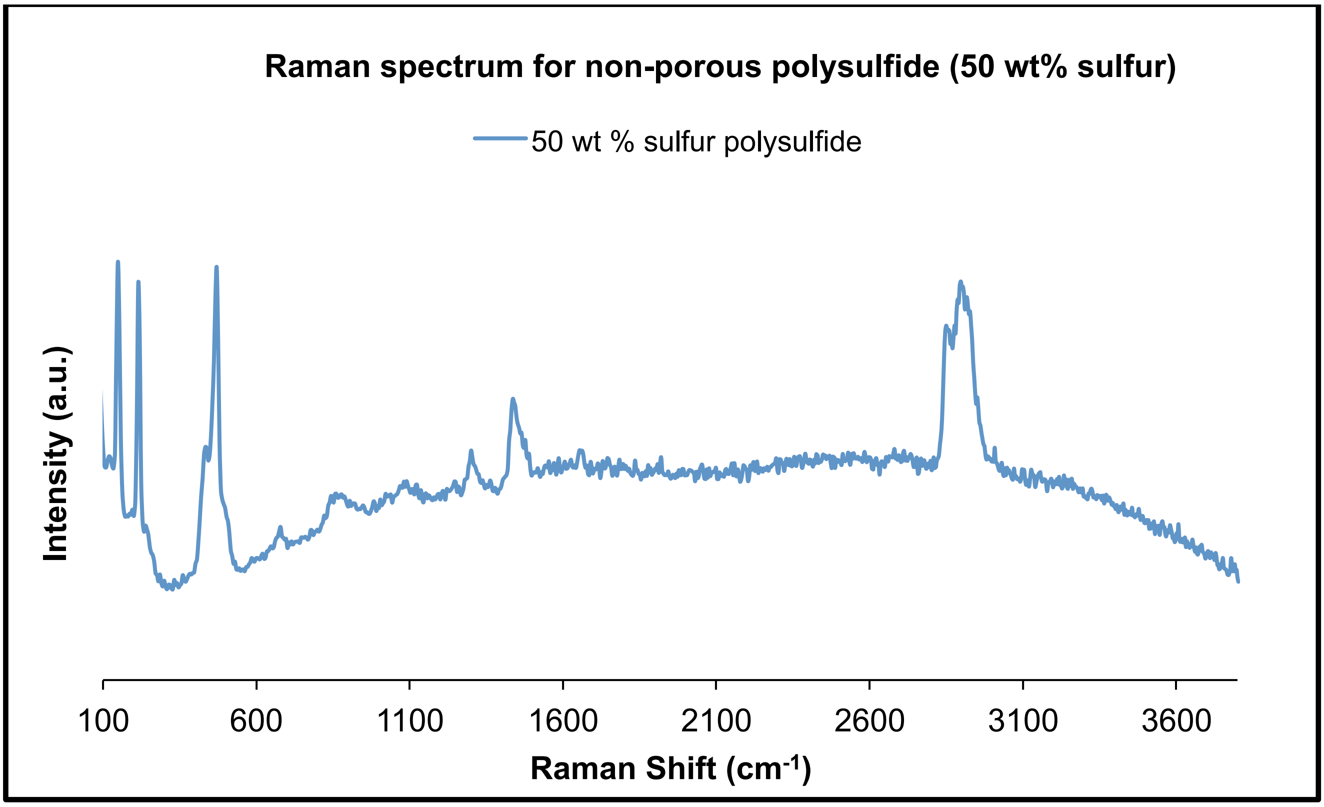

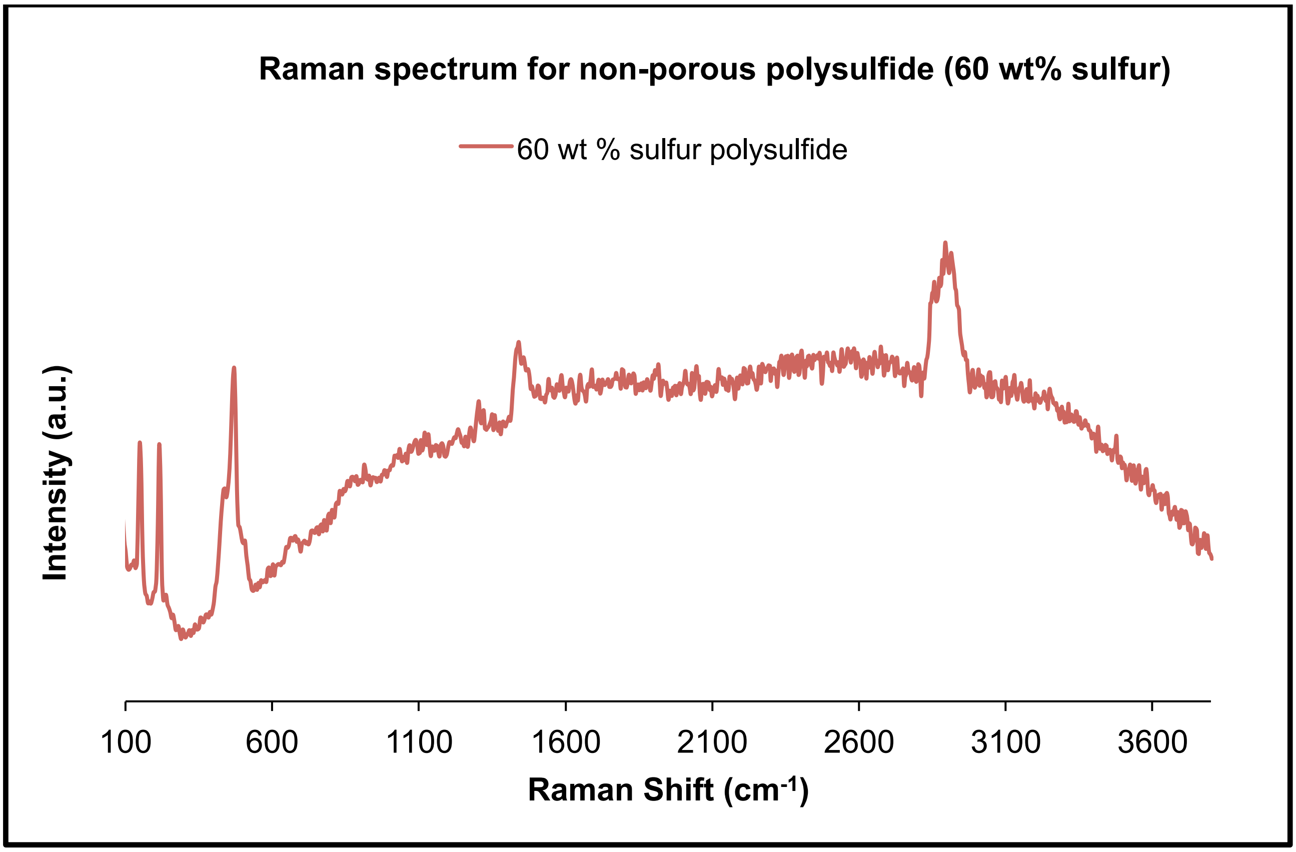
Raman spectrum for non-porous polysulfide (60 wt% sulfur)
60 wt % sulfur polysulfide
Intensity (a.u.)
100
600
1100
1600
2100
2600
3100
3600
Raman Shift (cm-1)

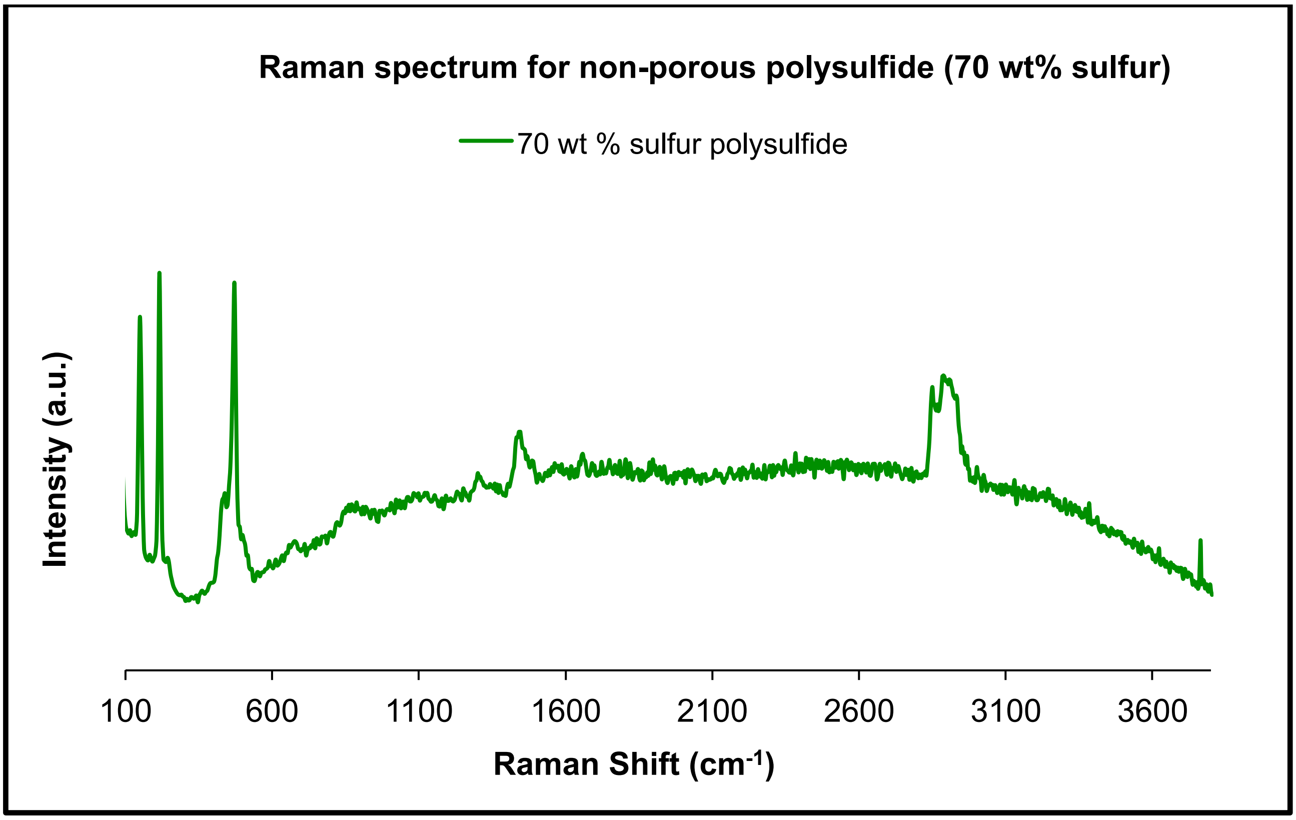
Raman spectrum for non-porous polysulfide (70 wt% sulfur)
70 wt % sulfur polysulfide
Intensity (a.u.)
100
600
1100
1600
2100
2600
3100
3600
Raman Shift (cm-1)

Figure S4: Differential Scanning Calorimetry (DSC) for non-porous canola oil polysulfides

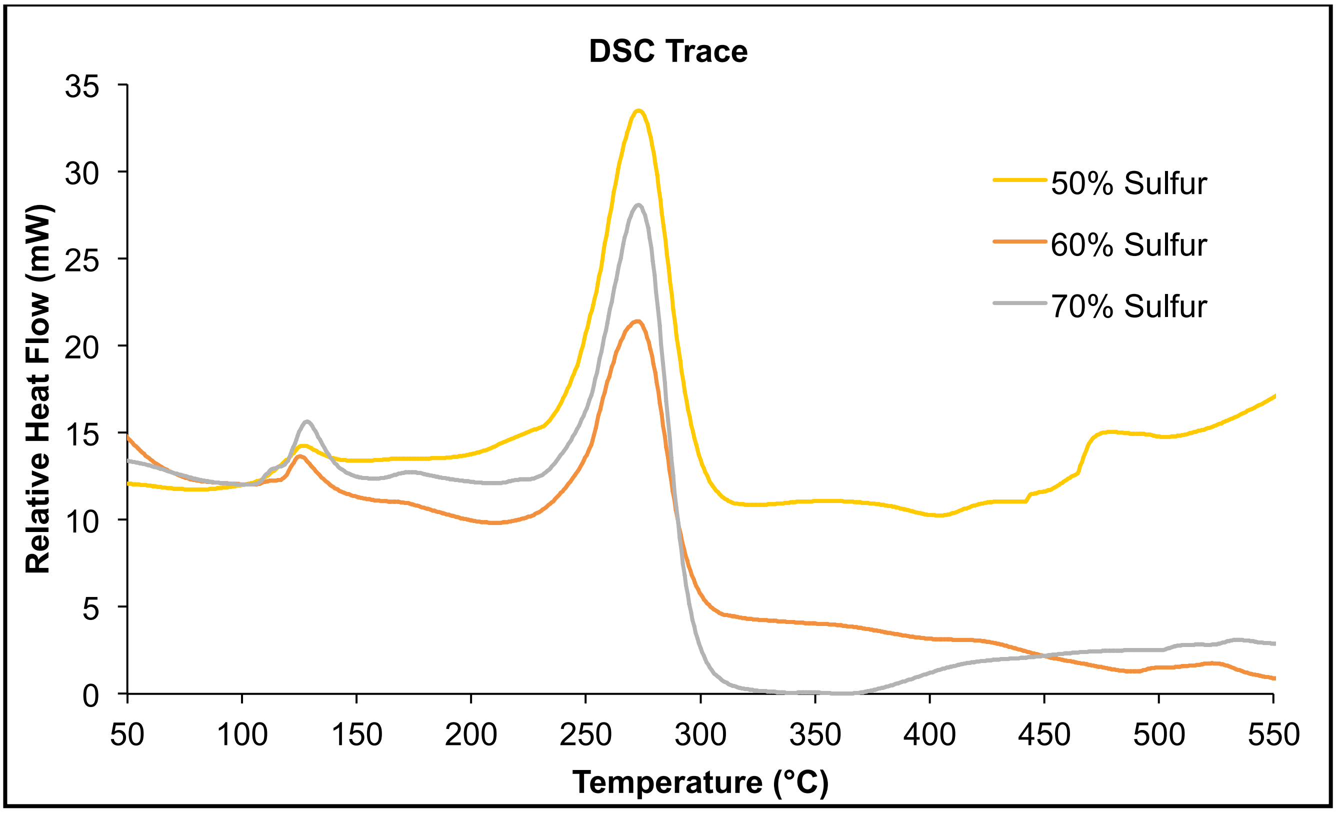

Figure S5: Thermogravimetric Analysis (TGA) for non-porous canola oil polysulfides

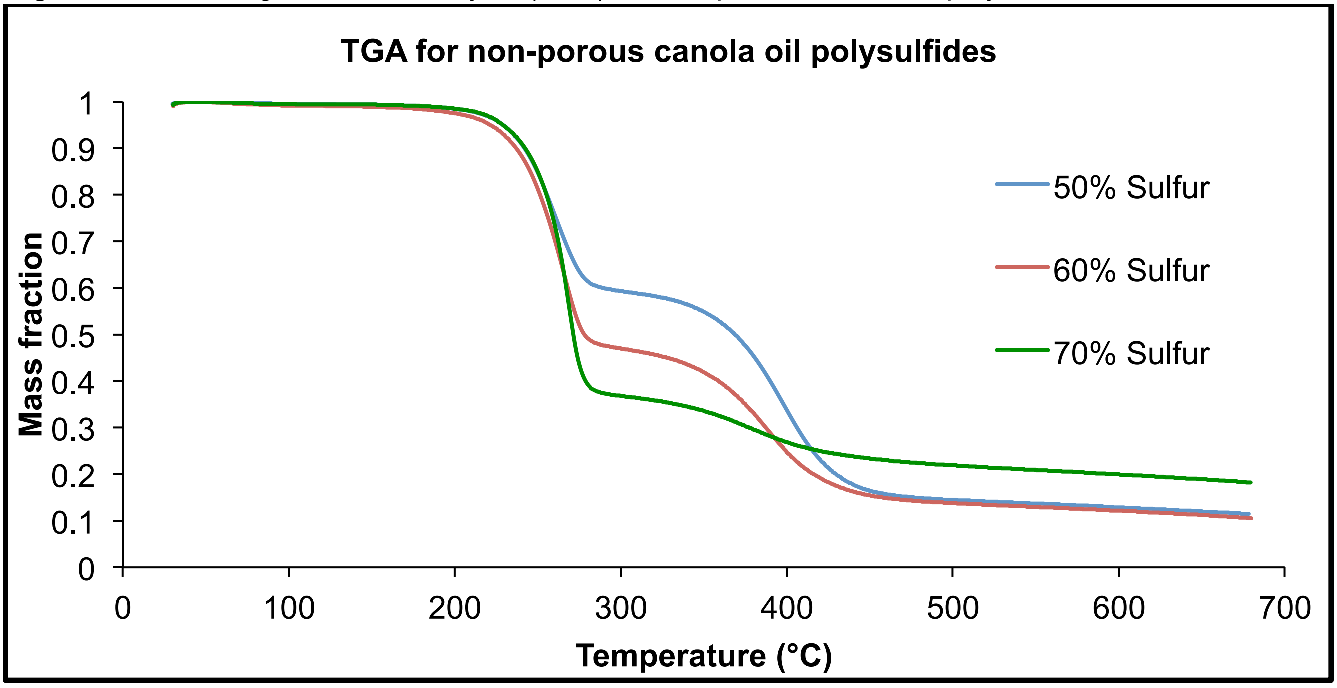

Figure S6: Representative SEM images for non-porous canola oil polysulfide (50% sulfur)

Note that both smooth surfaces and particles are observed. This is consistent with having some particulate sulfur or polysulfides bound to the surface. This is thought to form upon milling the polymer. Cutting a cross-section results in a smooth surface.

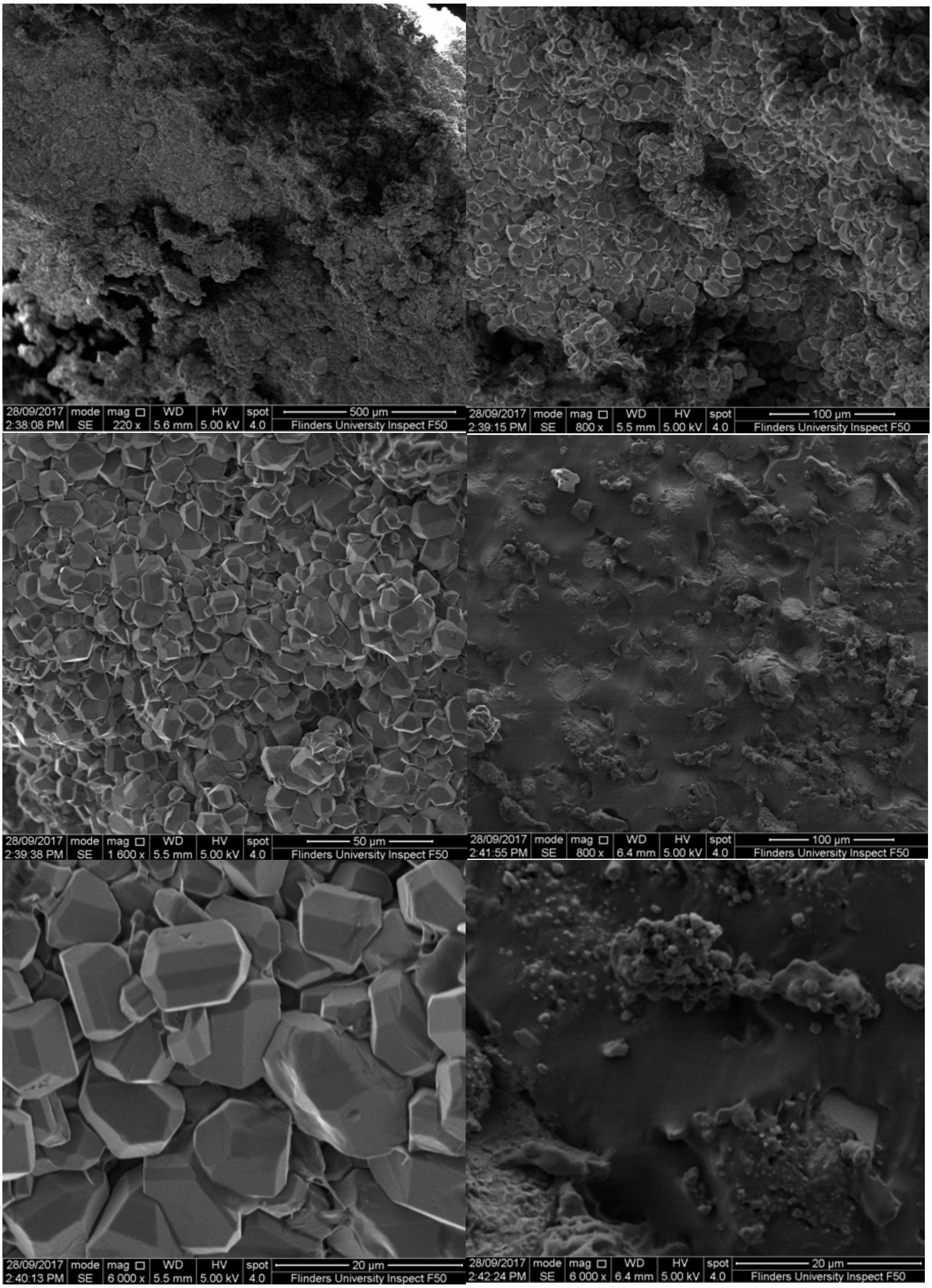

Figure S7: Representative SEM images for non-porous canola oil polysulfide (60% sulfur)

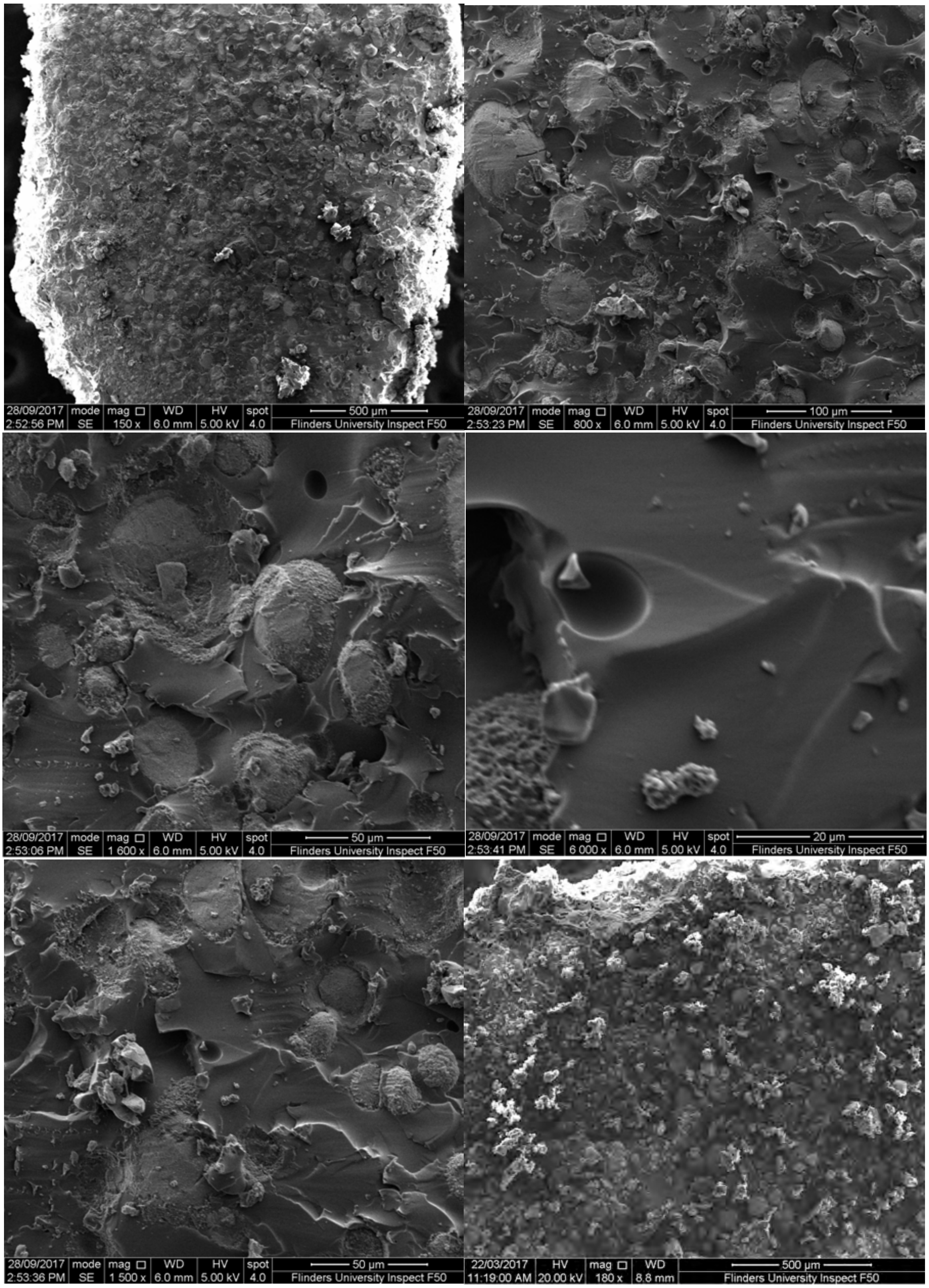

Figure S8: Representative SEM images for non-porous canola oil polysulfide (70% sulfur)

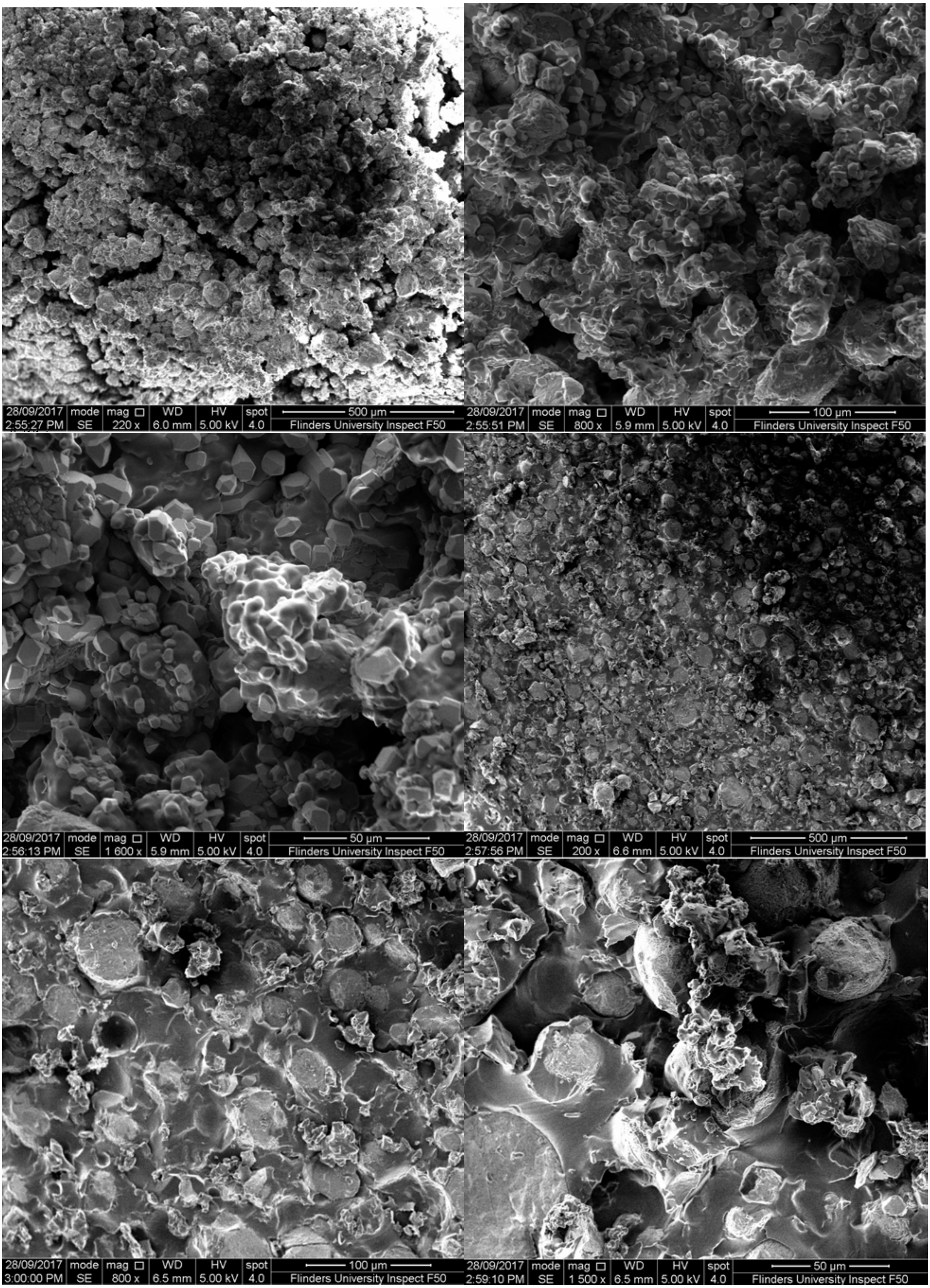

Fe(III) calibration curve for UV-Vis analysis

A solution of $FeCl_3$ was prepared by adding 50 mg $FeCl_3$ to a 1 L volumetric flask and then adding water up to 1 L mark. The solution was then equilibrated at room temperature for 48 hours. The pH of this solution was measured to be 3.04, using a pH meter. Samples of concentrations varying from 0.2 mg/L Fe(III) to 50 mg/L were then prepared and used to construct a calibration curve based on the absorbance at 306 nm. The calibration curve is shown below. This calibration curve was used to monitor removal of Fe(III) from solution in subsequent experiments.

Figure S9: Calibration curve for aqueous $FeCl_3$

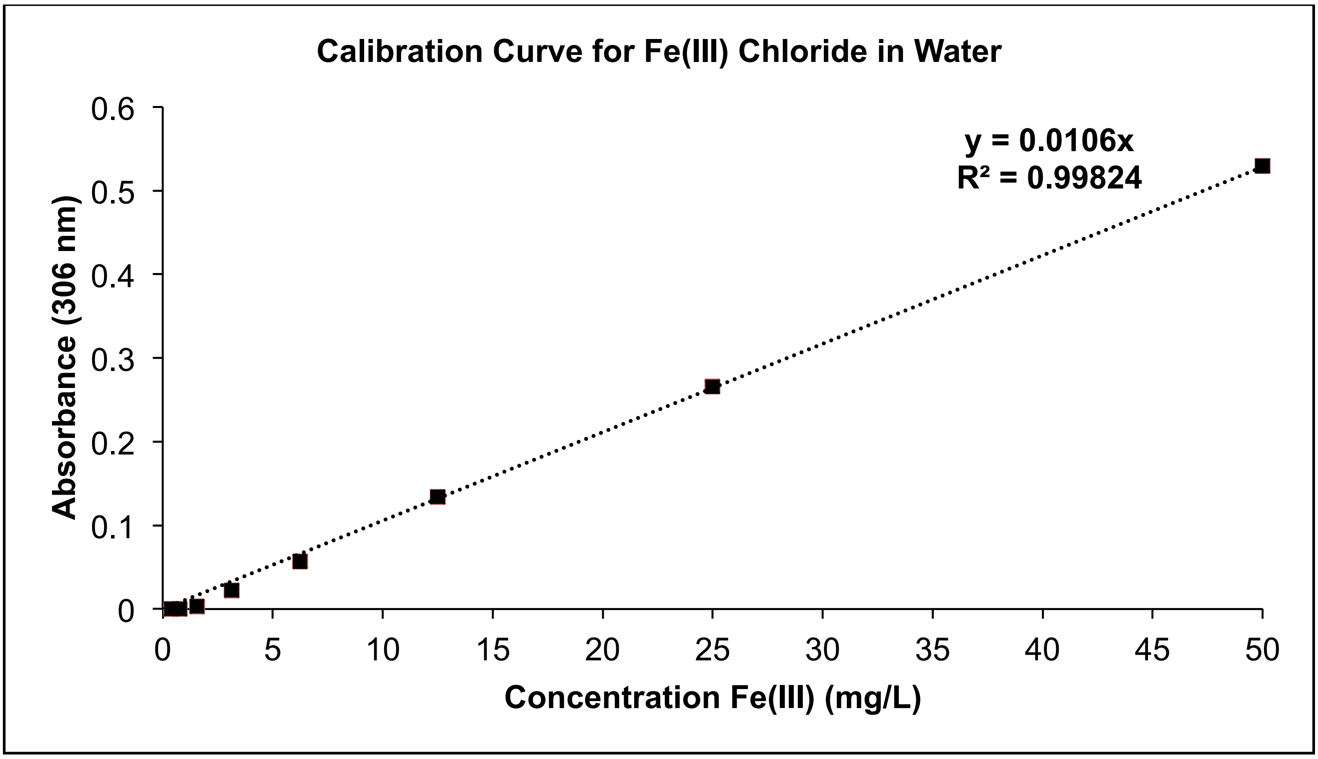

Fe(III) removal from water using non-porous polysulfides of varying sulfur content

A 20 mL aliquot of the 50 mg/L $FeCl_3$ solution was added to each of 12 centrifuge tubes (50 mL tube). Three samples served as control experiments in which no polymer was added. To the remaining samples, 2.0 g of the polysulfide was added: 3 samples were treated with the 50% sulfur polymer, 3 samples were treated with the 60% sulfur polymer, and 3 samples were treated with the 70% sulfur polymer. The particles size for all polymer samples was 1.0-2.5 mm. All samples were then placed on a rotating mixer (25 RPM) at room temperature. The concentration of Fe(III) was monitored for all samples by taking a 1 mL aliquot and removing any solids using a benchtop centrifuge. The absorbance of the supernatant at 306 nm was then recorded for the sample. The measurements were recorded at 24 and 48 hours. The concentration of Fe(III) in solution over time is plotted below. Error bars indicate ± 1 standard deviation from the mean value for the triplicate experiments. After 24 hours, the iron concentration in solution was typically between 3 and 6 mg/L. There was no significant difference between the 50, 60 and 70% sulfur polymers under these conditions.

Figure S10: Removal of Fe(III) from water using the non-porous polysulfides

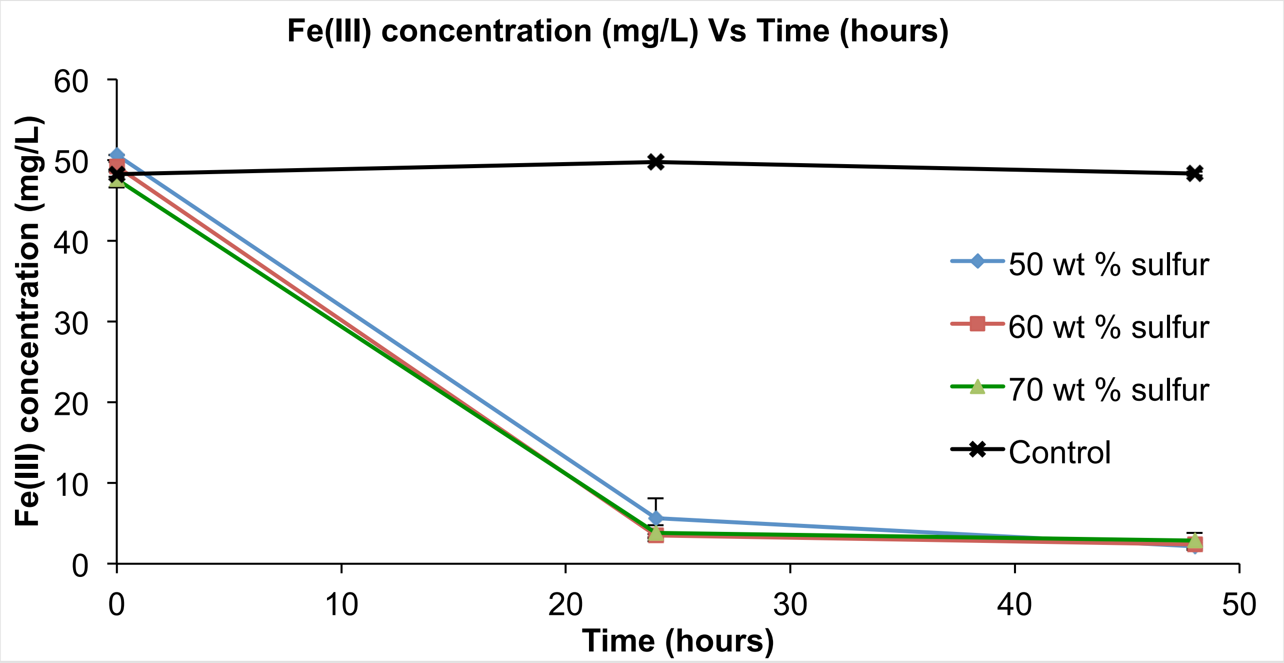

Fe(III) removal from water using varying amounts of the 50% sulfur non-porous polysulfide
The non-porous polysulfide prepared at 50% sulfur and 50% canola oil was used for these experiments.

The stock solution of $FeCl_3$ was prepared at 50 mg/L and equilibrated for 48 hours. This solution had a pH = 3.0, as prepared. Various masses of the non-porous polysulfide (50% sulfur) were added to 20 mL samples of the $FeCl_3$ solution (run in triplicate for all experiments). The particle size of the polymer was 1.0-2.5 mm. The samples were incubated on a rotary mixer at room temperature for 24 hours, as described in the previous iron capture experiment. The absorbance was monitored at 306 nm to determine iron concentration for all samples. The results are summarised in the table below, with 2.0 grams of non-porous polysulfide (50% sulfur) required to reduce the concentration of Fe(III) in water below 10 mg/L

Figure S11: 2.0 grams of non-porous polysulfide (50% sulfur) is required to reduce the concentration of Fe(III) in water below 10 mg/L**.** The control sample does not contain polymer.

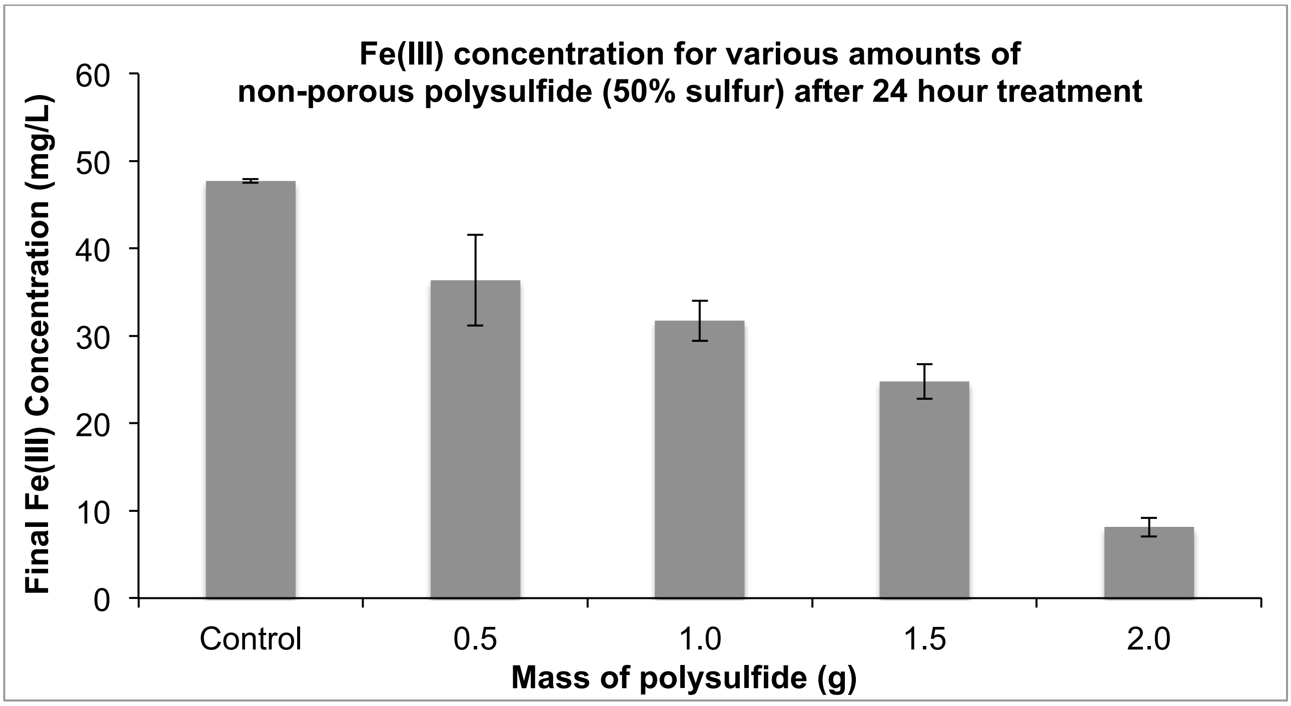

Optimised treatment process
(45 mg/L $FeCl_3$, pH 3, 2.0 grams non-porous polysulfide, 50% sulfur)

The stock solution of $FeCl_3$ was prepared at 45 mg/L and equilibrated for 48 hours. This solution had a pH = 3.0, as prepared. 2.0 grams of the non-porous polysulfide (50% sulfur) was added into 20 mL of $FeCl_3$ solution (triplicate experiments). The particle size was 1.0-2.5 mm. Another three tubes contained only Fe(III) chloride and no polymer were used as controls. The samples were incubated on a rotary mixer at room temperature for 24 hours, as described in the previous iron capture experiment. The absorbance was monitored at 306 nm to determine iron concentration for all samples. The results are summarised in the table below:

Sample	Initial Concentration (mg/L)	Final Concentration (mg/L)
Treatment	45.2 ± 0.2	3.70 ± 0.02
Control	45.5 ± 0.4	45.4 ± 0.01

Figure S12: Optimised Fe (III) removal from water using non-porous polysulfide (50 wt% sulfur)

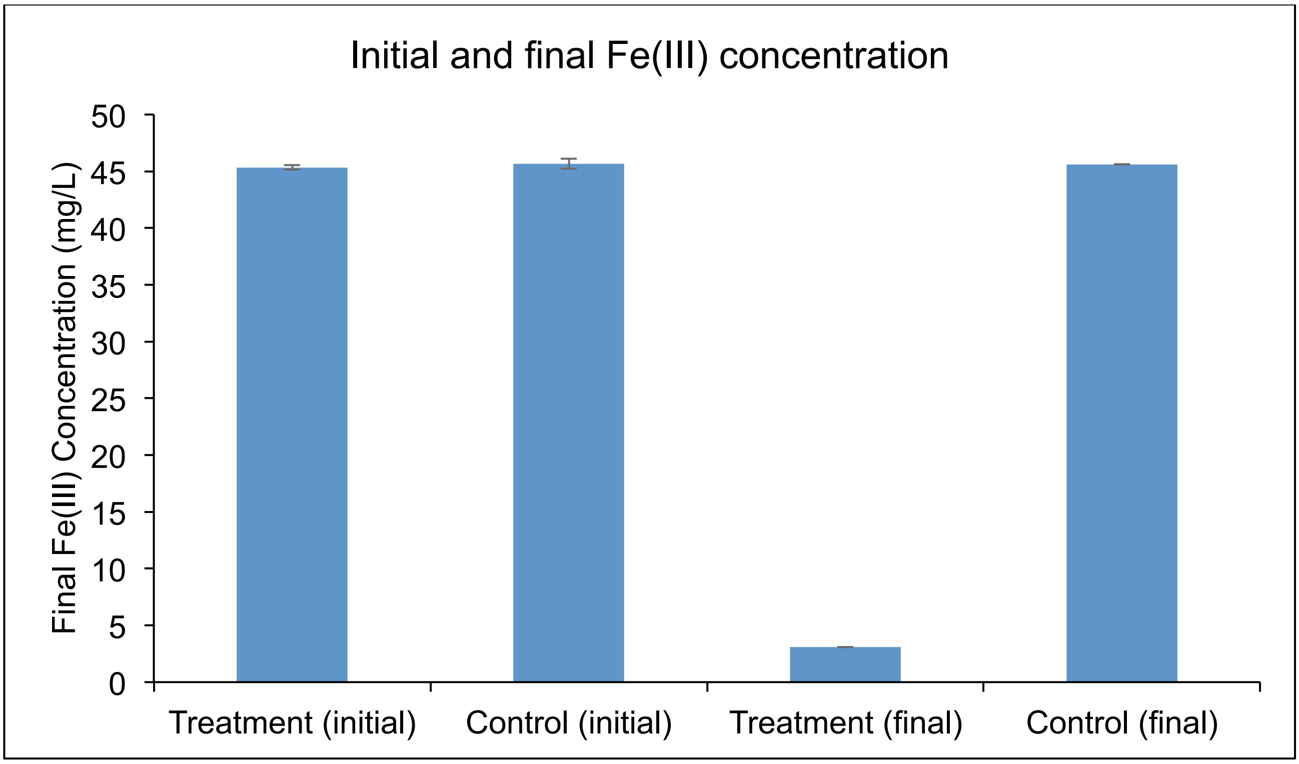

Figure S13: After the optimised Fe(III) removal treatment, the water no longer appears coloured. Left: $FeCl_3$ (45 mg/L) and Right: Water after treatment with non-porous polysulfide (24 hours, 50 wt% sulfur) this water sample originally contained 45 mg/L Fe(III).

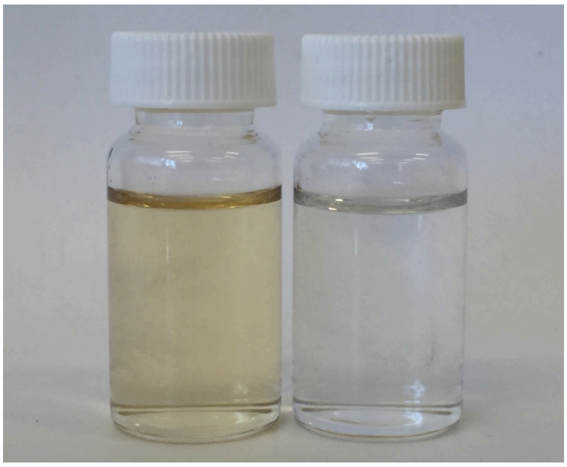

The polysulfide used in the previous experiment was isolated by filtration and dried thoroughly before analysing by SEM and XRD. The surface of the non-porous polysulfide (50 wt% sulfur) did not appear to change after the iron treatment process. The XRD pattern was highly similar after the Fe(III) treatment. We attribute these results to the relatively low absolute amount of iron bound to the polymer (< 0.5 mg Fe(III) per g non-porous polysulfide) which would not be expected to strongly alter the surface structure of the polymer.

Figure S14: SEM analysis of polysulfide (50% sulfur) after exposure to $FeCl_3$ solution

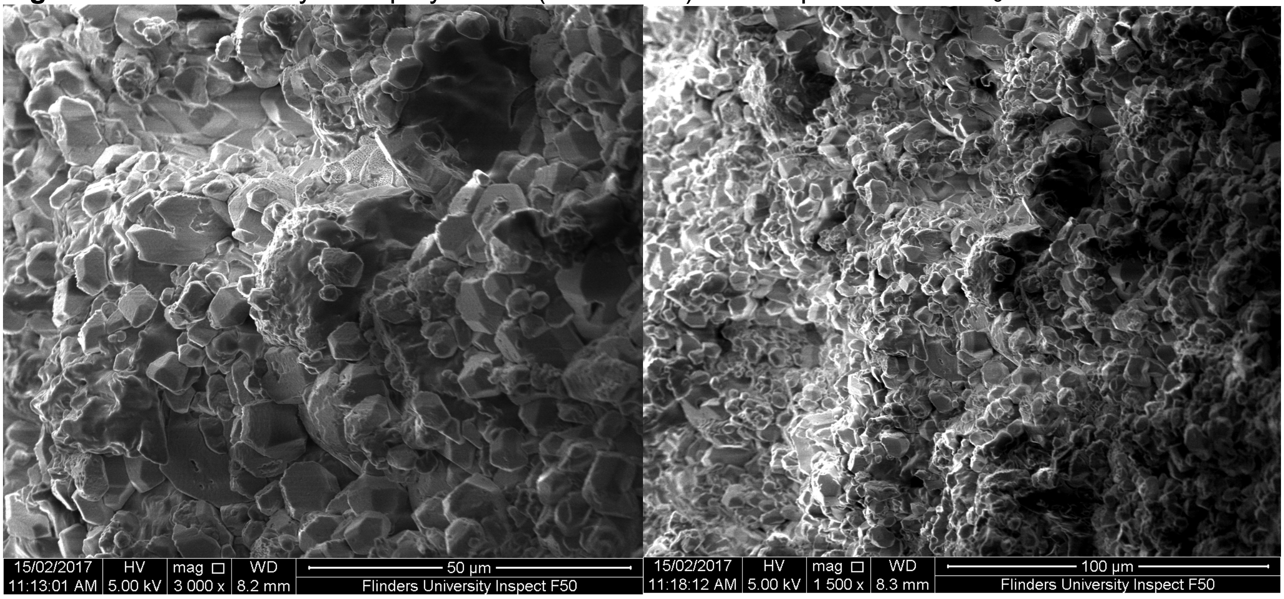

Figure S15: XRD before and after iron (III) treatment

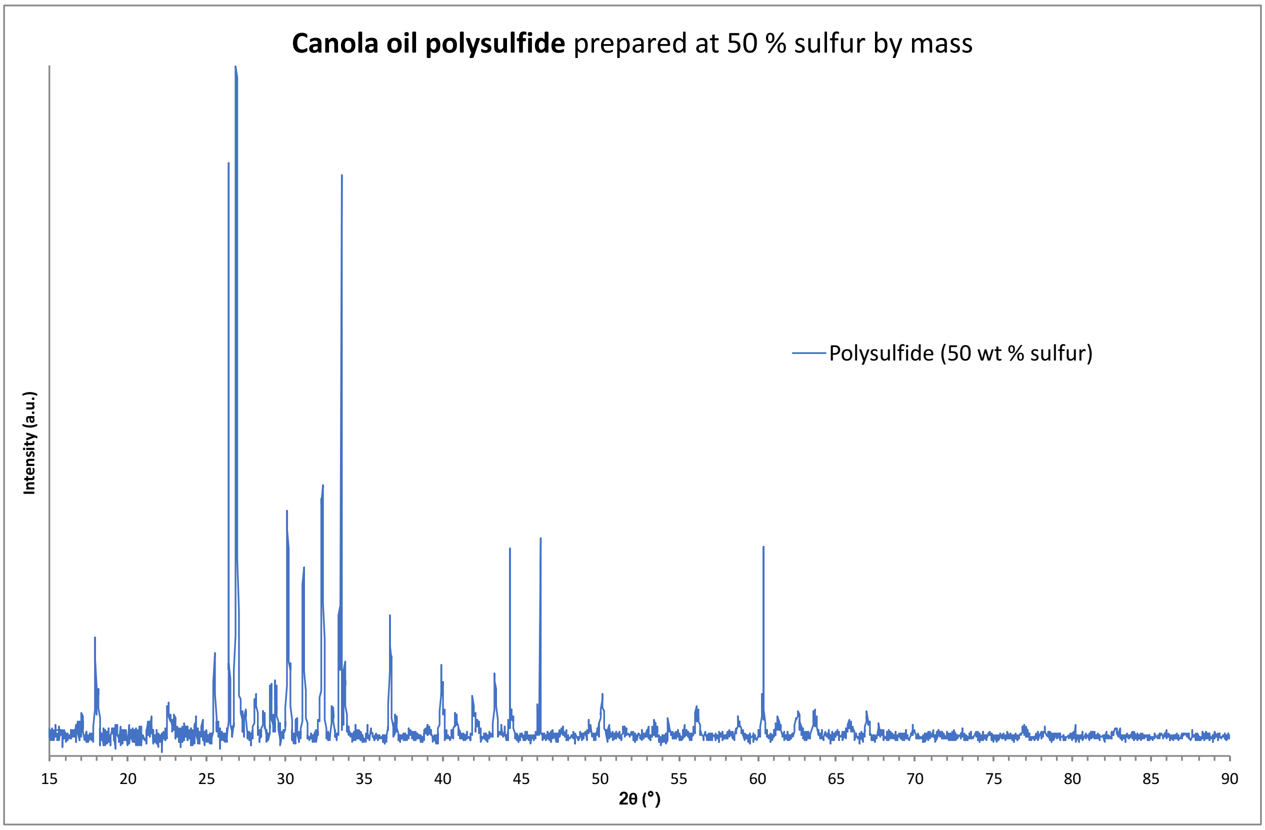

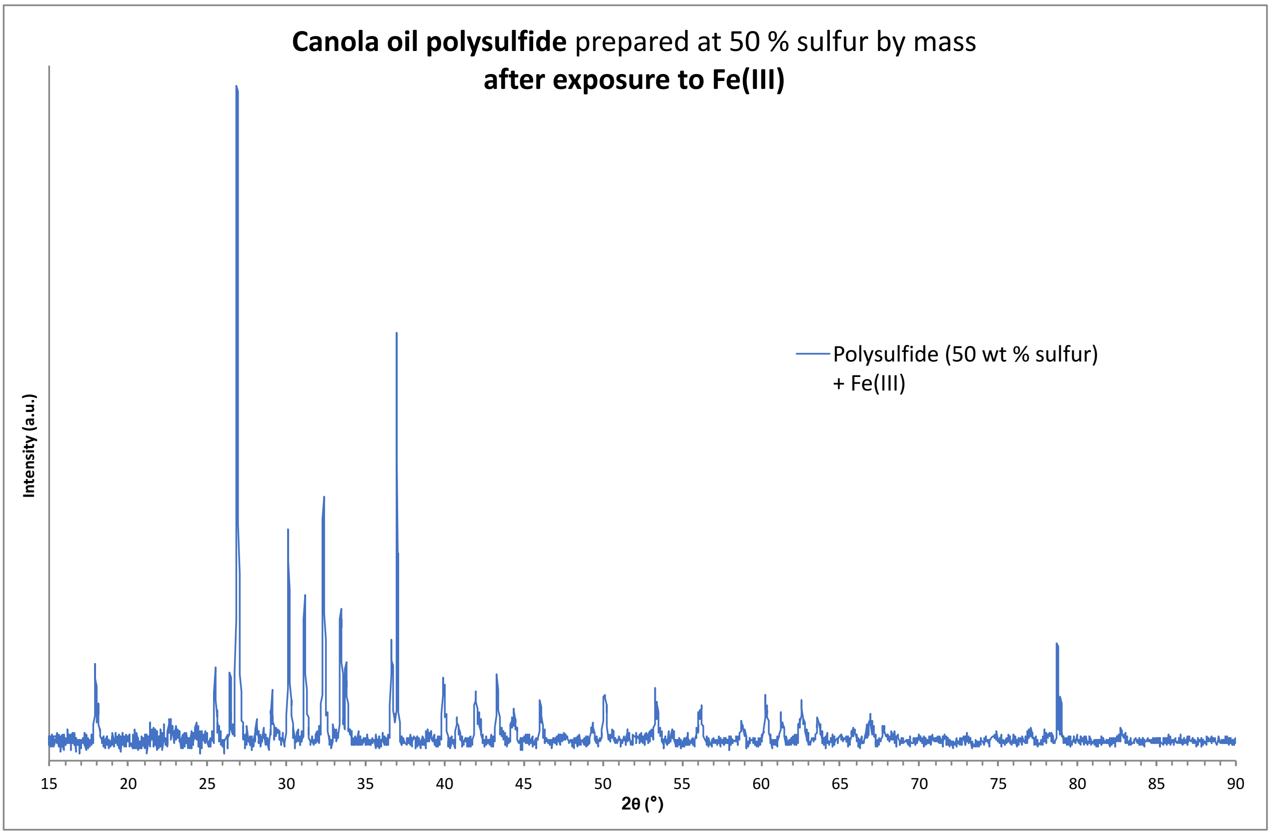

Treatment of non-porous polysulfide (50 wt% sulfur) with hydrogen peroxide

1.0 g of non-porous polysulfide (50 wt% sulfur) was added to 20 mL samples of 15% H_2O_2 (with triplicate samples for all experiments). The samples were incubated on a rotary mixer at room temperature for 24 hours. The polysulfide was then filtered, washed with 3 x 40 mL of water and dried in air overnight. SEM and EDS was then used to analyse the surface of the hydrogen peroxide treated polysulfide.

Figure S16: Image of non-porous polysulfide (50 wt% sulfur) after treatment with hydrogen peroxide. The surface appears to be "bleached" and slightly lighter in colour.

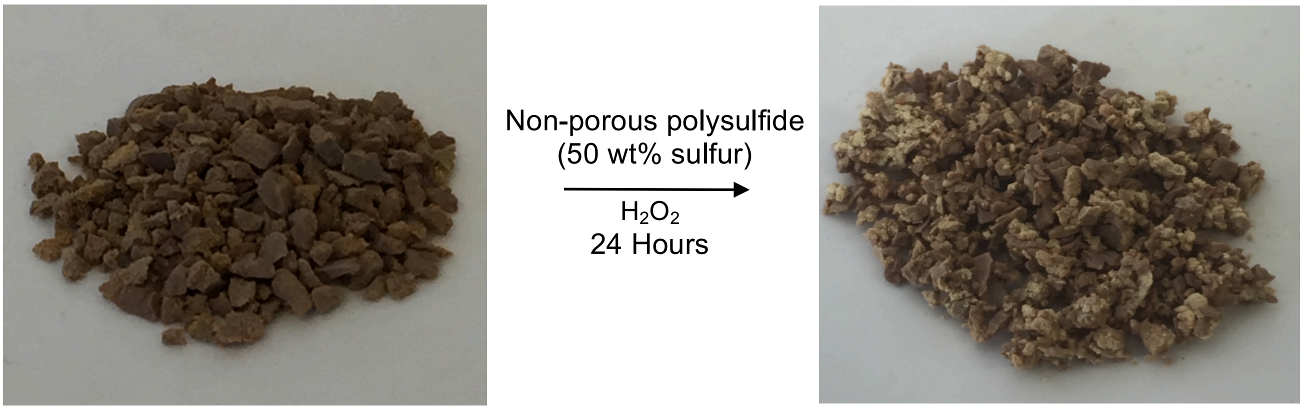

SEM/EDS Analysis

Figure S17: SEM and EDS analysis of non-porous polysulfide (50 wt% sulfur) after treatment with hydrogen peroxide.

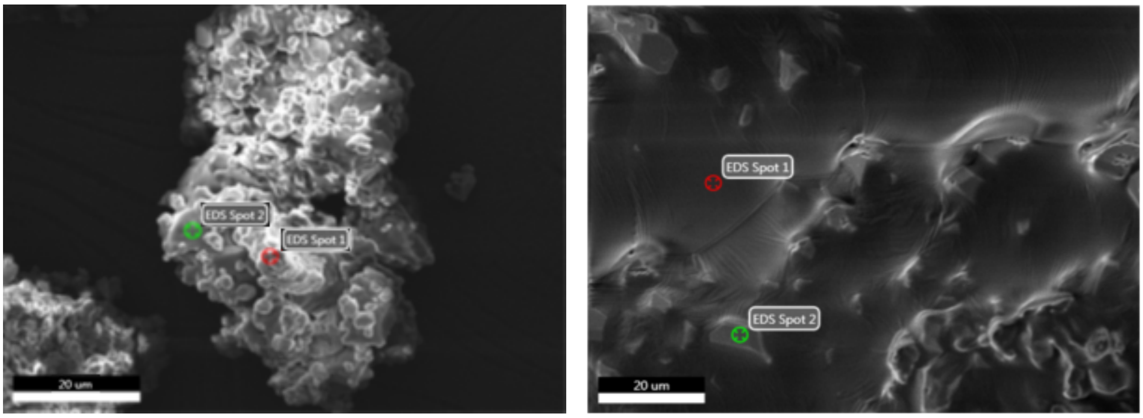

SEM/EDX results for before (left) and after (right) 24-hour hydrogen peroxide treatment

Atom	Weight % (Before)	Weight % (After)
Sulfur	36.54	29.7
Carbon	57.72	63.3
Oxygen	3.04	3.2

EDS results for spot 1 in both SEM images

Removal of Fe(III) from water using a non-porous polysulfide (50 wt% sulfur) previously treated with H_2O_2

The stock solution of $FeCl_3$ was prepared at 50 mg/L at a pH of 3.0 as described previously. The H_2O_2 treated polysulfide (2.0 g non-porous polymer, 50 wt% sulfur) was added to 20 mL samples of the $FeCl_3$ solution. The samples were incubated on a rotary mixer at room temperature for 24 hours, as described in the previous iron capture experiment. The absorbance was monitored at 306 nm to determine iron concentration for all samples. Control experiments with no polymer and also 2.0 g of polymer that was not treated with H_2O_2 were also used for comparison. The results are summarized in the table below. The prior treatment with H_2O_2 does not impair the polymer's ability to capture Fe(III).

Sample	Initial Fe(III) concentration (mg/L)	Final Fe(III) concentration
Non-porous polysulfide (H_2O_2 treated)	45.2 ± 0.1	3.1 ± 0.1
Non-porous polysulfide (No H_2O_2)	46.2 ± 0.1	4.9 ± 0.2
Control (iron)	46.1 ± 0.1	44.7 ± 0.6

Figure S18: Prior treatment of non-porous polymer with 15% aqueous hydrogen peroxide does not impair its ability to remove Fe(III) from water.

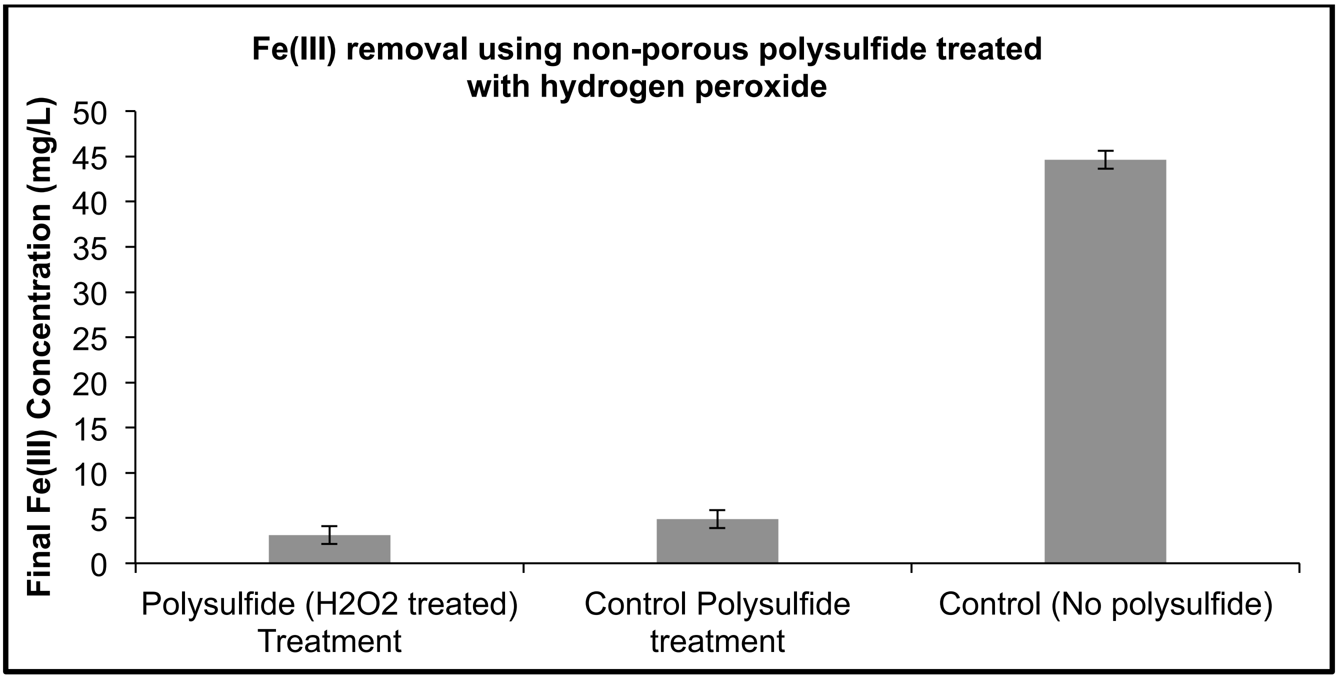

Litre-scale Fe(III) removal from water using the non-porous polysulfide (50 wt% sulfur)

The stock solution of $FeCl_3$ was prepared at 50 mg/L as described previously. This solution had a pH = 3.0, as prepared. 200 g of the polysulfide (50% sulfur) was added to 1 L of $FeCl_3$ solution. The particle size of the polymer was 1.0-2.5 mm. The solution was stirred at room temperature for 24 hours, after which time the polymer was removed by filtration. The absorbance was monitored at 306 nm to determine Fe(III) concentration. Atomic Absorption Spectroscopy was used as an independent measurement of total iron concentration. The results are tabulated below, along with an image of the water sample before and after treatment.

Fe(III) concentration measured by UV-Vis:

Sample	Average Concentration (mg/L)
Initial (UV/Vis)	52.2 ± 1.4
Final (UV/Vis)	1.31 ± 0.01

Total iron concentration measured by AAS:

Sample	Average Concentration (mg/L)
Initial (AAS)	49.5 ± 1.0
Final (AAS)	1.29 ± 0.06

Figure S19: Image of water sample (50 mg/L in iron) before and after treatment with the non-porous polysulfide (50 wt% sulfur).

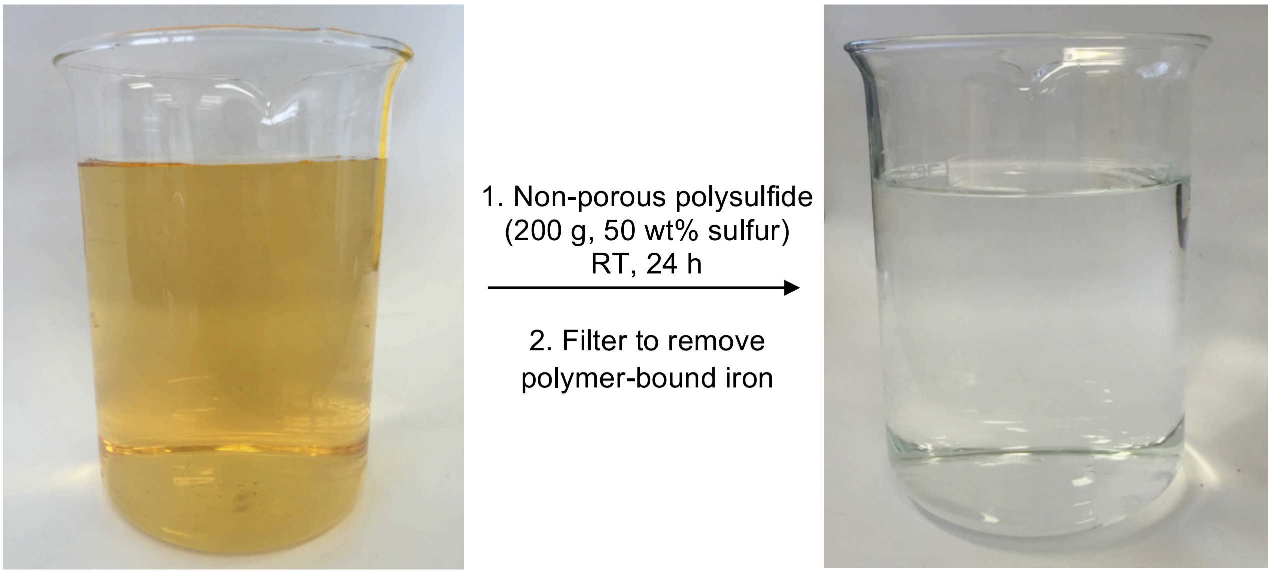

Figure S20: Mixing elemental sulfur in water (left) results in caking that is inconvenient for batch processing and filtration. The yellow solid adhere to the walls of the tube. In contrast, the non-porous polysulfide (50 wt% sulfur) does not cake in water and can be easily removed by filtration.

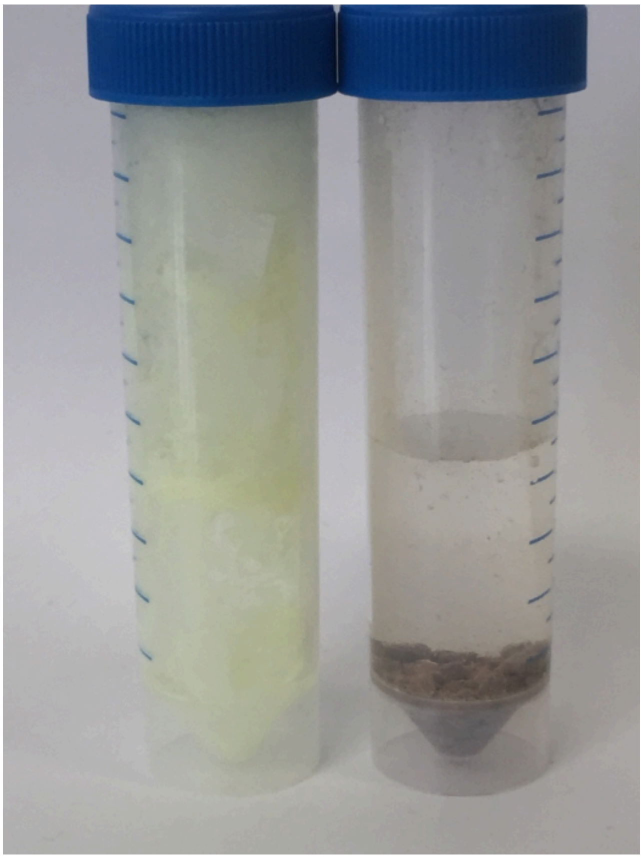

Fe(II) removal from water using the non-porous polysulfide (50 wt% sulfur)

The stock solution of $FeCl_2$ was prepared at 50 mg/L. 20 mL of this solution was transferred into 6 x 50 mL centrifuge tubes. A pH meter was used to determine the initial pH of the solution. 2.00 grams of non-polysulfide were added to three of the solutions, while the remaining three solutions were controls to which no polymer was added. All samples were then placed on a rotating mixer (25 RPM) at 23 °C for 6 hours. After 6 hours, the pH of each solution was recorded using a pH meter and 1 mL aliquots of each solution were transferred into a centrifuge tube. Any remaining solids were then removed using a bench top centrifuge. The concentrations of these samples were then monitored by atomic absorption spectroscopy (AAS). The results are shown below in Figure S27.

pH (before treatment)	pH (after treatment)
5.92	5.65

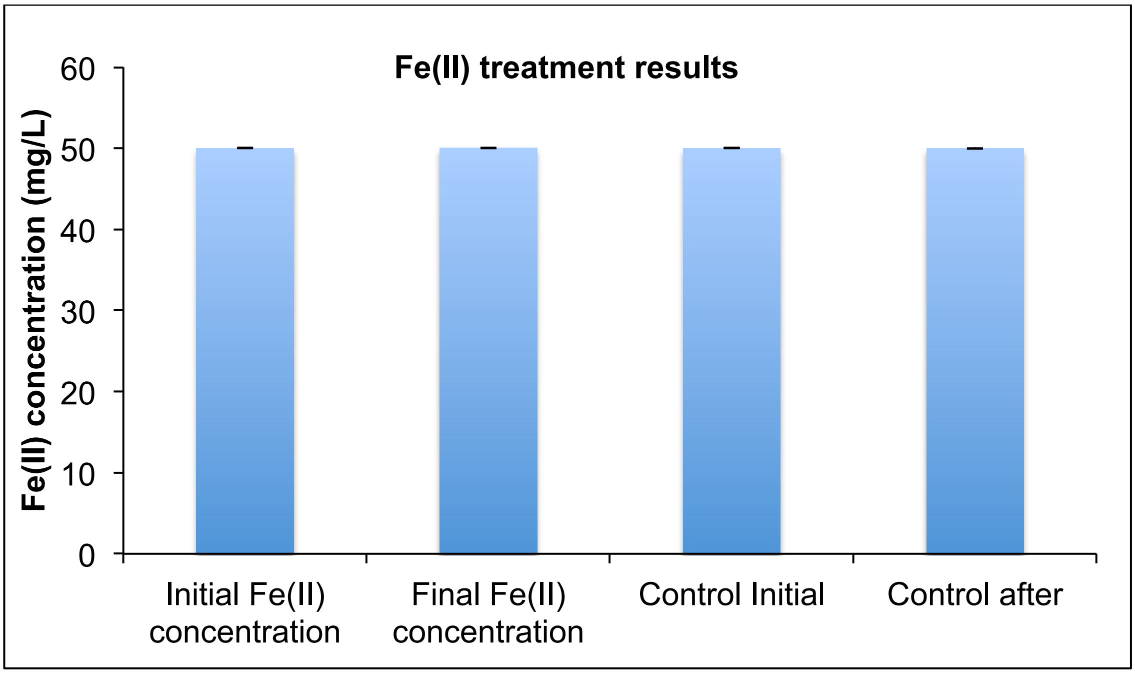

Figure S21 - Fe(II) sorption test using 2.00 g of the non-porous polysulfide in 20 mL of Fe(II). Negligible amounts of iron were bound to the polymer

Porous canola oil polysulfide synthesis (50 wt% sulfur)

NaCl (14.0 grams) was ground into a fine powder using a mortar and pestle. Sulfur (technical grade, 3.00 g) was added to a 250 mL round bottom flask and then melted, with stirring, before heating further to 180 °C. Canola oil (3.00 g) was added drop wise over 2 minutes. The reaction mixture was stirred at a rate that ensured efficient mixing of the two phases. The NaCl powder was then added over 5-10 minutes, and the stirring rate was continually adjusted to ensure efficient mixing. Heating was continued for an additional 10-15 minutes at 180 °C, over which time the reaction mixture formed a brown solid. The reaction was cooled to room temperature and removed from the flask. The product (20.0 g) was milled for 1 minute in a blender (8.0 cm rotating blade) to give various particle sizes (typically between 0.1 mm and 3.0 cm). The particles were then transferred to a beaker and washed with 150 mL of water for 1 hour with stirring. The washing process was repeated at least one more time to remove as much sodium chloride as possible. Drying in a desiccator under vacuum provided the porous polymer as a soft rubbery sponge. If sodium chloride was visible as a white solid on the surface of the polymer, the washing and drying steps were repeated until a constant mass was obtained. Near quantitative yields (>5.90 g, >98%) were typically obtained in this process.

Figure S22: Porous canola oil polysulfide (50 wt% sulfur) particles of size ranges: **A)** 1.0-2.5 mm, **B)** 2.5-5 mm, **C)** >5.0 mm.

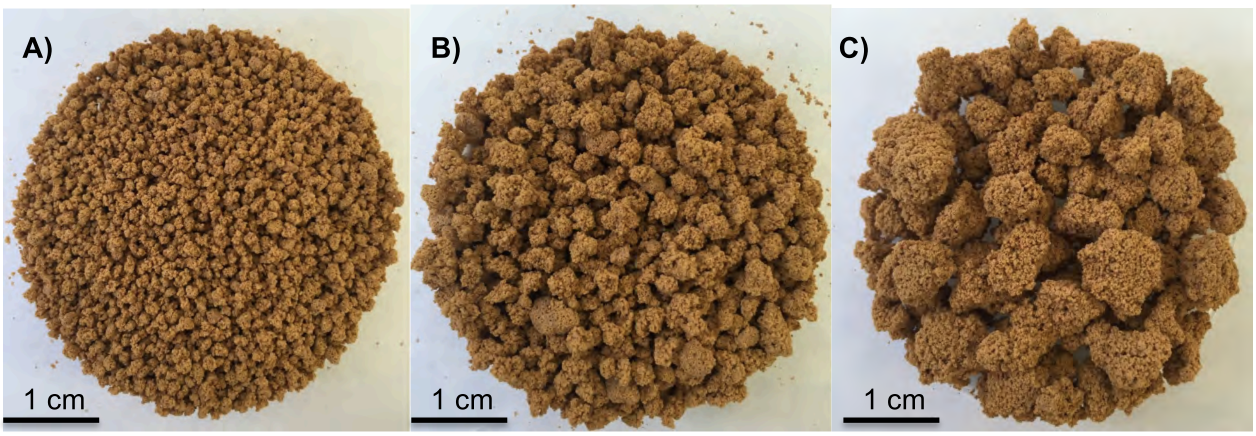

Figure S23: Representative SEM images of porous canola oil polysulfide (50 wt% sulfur). For a random sample of 50 pores, the diameter of the pore was found to be 119 ± 53 μm.

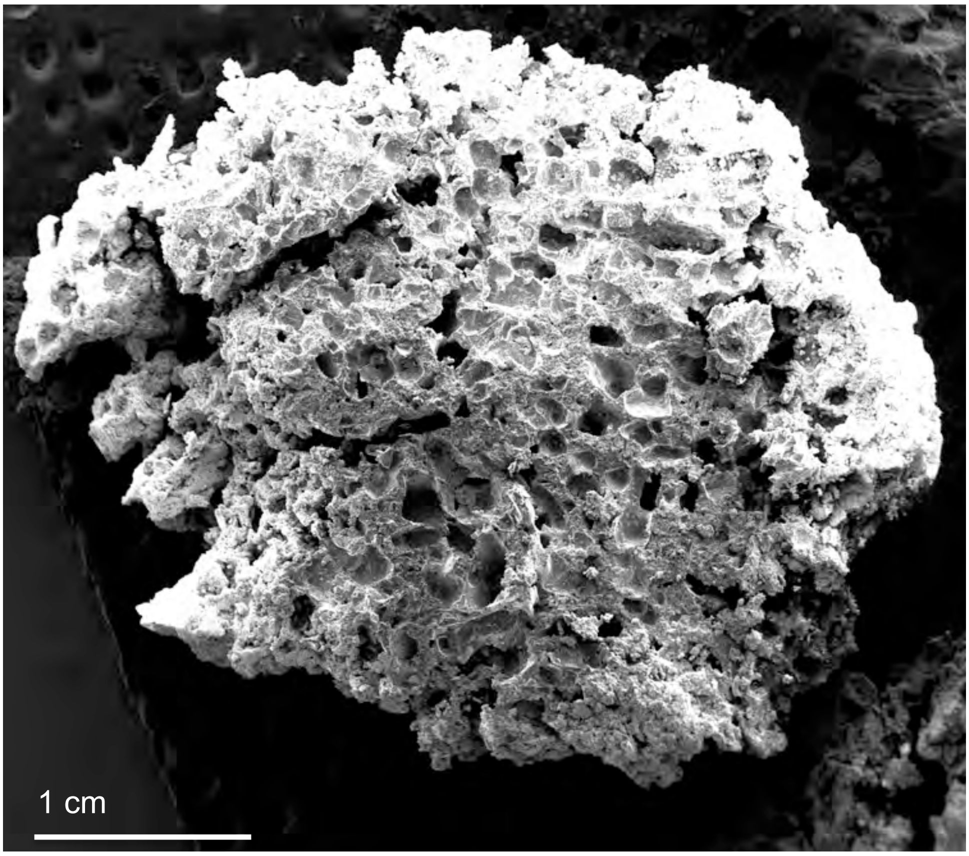

Figure S24: IR spectrum of the porous canola oil polysulfide (50 wt% sulfur). The IR spectrum of the non-porous polysulfide is also plotted for comparison.

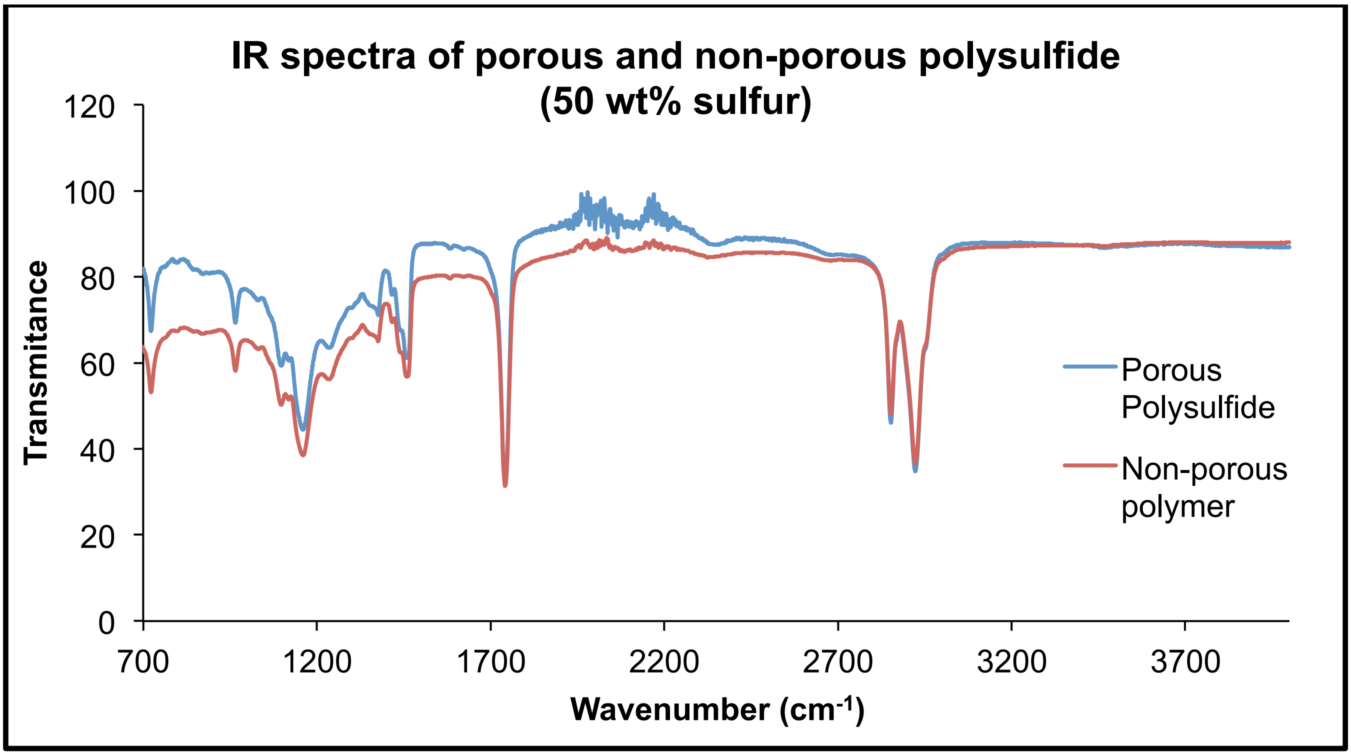

Figure S25: XRD of porous polysulfide (50 wt% sulfur). The XRD for the non-porous polysulfide (50 wt% sulfur is shown for comparison).

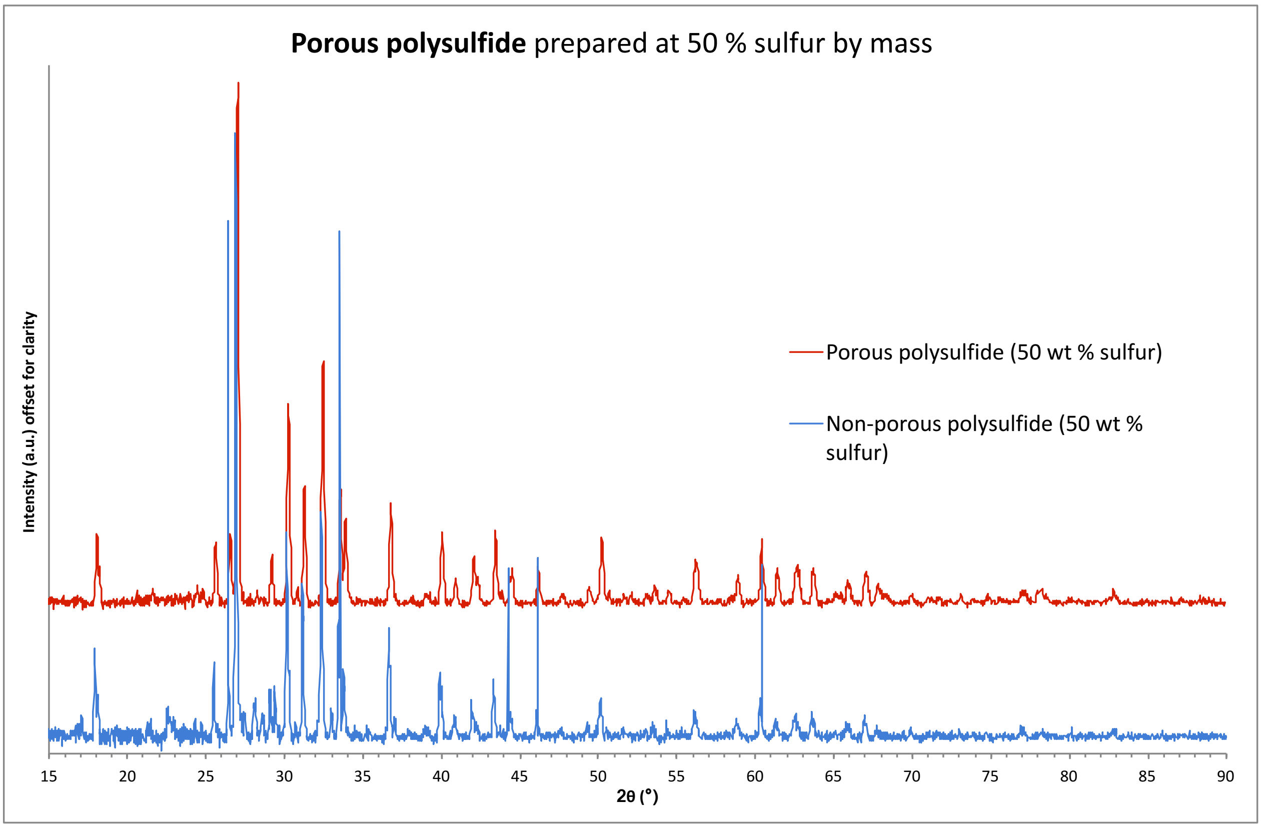

Stability of porous canola oil polysulfide at different pH

The stability of the porous canola oil polysulfide was tested in 3 solutions a different pH over a period of 7 days.

Solution 1 (pH ~13): 92 mg NaOH was dissolved in 2.31 mL D_2O to afford a 1 M solution of NaOH in D_2O. 600 µL of this solution was transferred to a separate vial containing 5.4 mL of D_2O, diluting the sample to 0.1 M NaOH. Finally, 30 µL of 1,4-dioxane was added as an internal standard. The pH was measured to be approximately 13 using a pH strip.

Solution 2 (pH ~1): 59.2 µL HCl (37% HCl in water) was added to 6 mL D_2O to afford a 0.1 M HCl solution. 30 µL dioxane was added as an internal standard. The pH was measured to be 1 by pH strip.

Solution 3 (pH ~6): 30 µL dioxane was added to 6 mL D_2O. The pH was measured to be 6 by pH strip.

2 mL of each solution was pipetted into separate glass vials containing 100 mg porous polysulfide. 2 mL of each solution were kept as controls. All solutions were incubated at 25 ºC for seven days. After this time, the resultant solutions were separated from the polymer and ^{1}H NMR spectrum of the solution was acquired. If the canola oil polysulfide hydrolysed, glycerol and fatty acids (e.g. oleic acid or sodium oleate) would be observed.

Results

At pH 1 for 7 days, the polymer did not break down to form any water-soluble material. The water was colourless over the experiment.

Figure S26: ^{1}H NMR spectra for experiment at pH 1 (control, no polymer): D_2O signal indicated at δ = 4.79 ppm and 1,4-dioxane at 3.73 ppm.

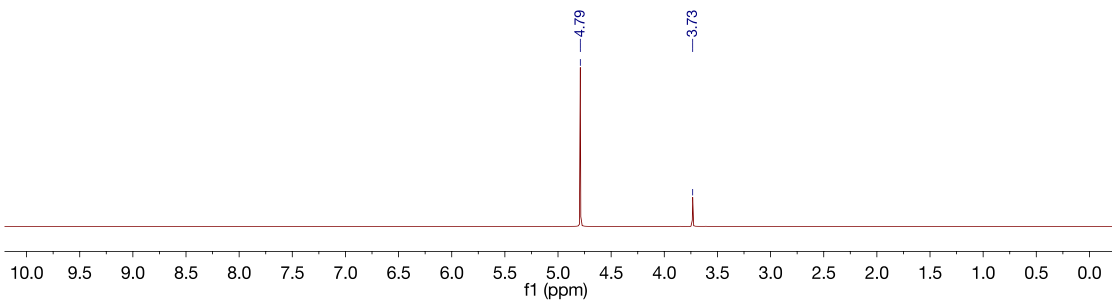

Figure S27: ^{1}H NMR spectra for experiment at pH 1 after 7 days with 100 mg polysulfide: only the D_2O signal at δ = 4.79 ppm and the 1,4-dioxane signal at 3.73 ppm were observed. No polymer decomposition products were detected in the water by ^{1}H NMR.

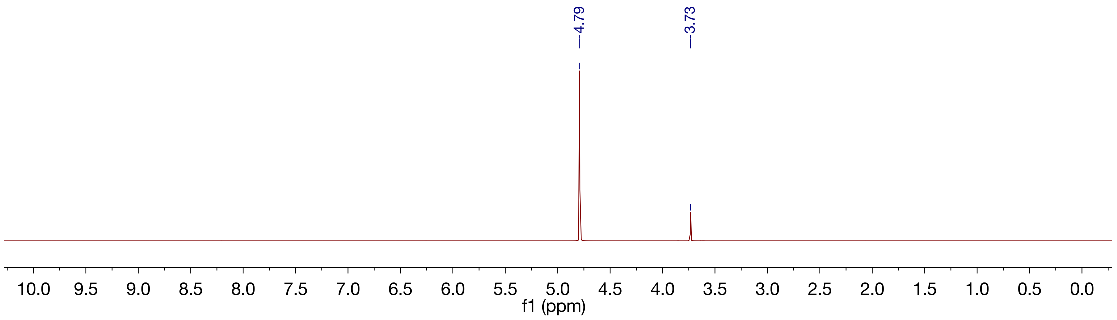

At pH 6 for 7 days, the polymer did not break down to form any water-soluble material. The water was colourless over the experiment. The polymer remained intact over the experiment.

Figure S28: ^1H NMR spectra for experiment at pH 6 (control, no polymer): D_2O signal indicated at δ = 4.79 ppm and 1,4-dioxane at 3.78 ppm.

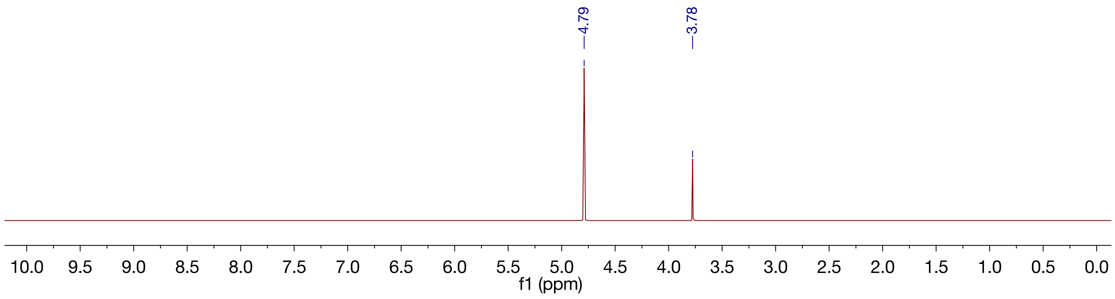

Figure S29: ^1H NMR spectra for experiment at pH 6 after 7 days with 100 mg polysulfide: only the D_2O signal at δ = 4.79 ppm and the 1,4-dioxane signal at 3.78 ppm were observed. No polymer decomposition products were detected in the water by ^1H NMR.

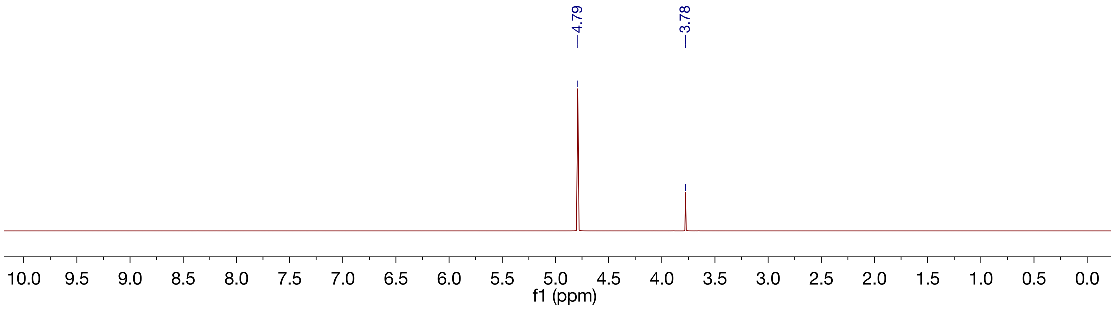

At pH 13 for 7 days, a slight colouration of the water was observed, but minimal organic material was observed by ^1H NMR and the polymer remained intact. Some signals did appear between 0.5 and 2.5 ppm, but these appear to only be trace amounts of polymer degradation products.

Figure S30: ^1H NMR spectra for experiment at pH 13 (control, no polymer): D_2O signal indicated at δ = 4.79 ppm and 1,4-dioxane at 3.76 ppm.

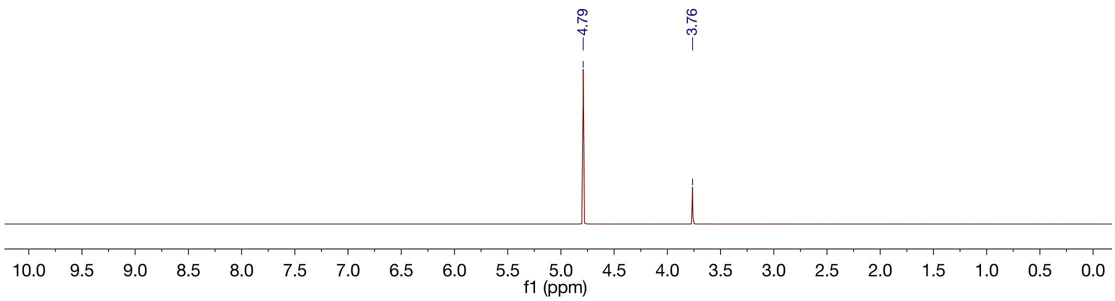

Figure S31: ^{1}H NMR spectra for experiment at pH 13 after 7 days with 100 mg polysulfide. Some trace amounts of polymer degradation product were observed between 0.5 and 2.5 ppm. These peaks are shown in the inset:

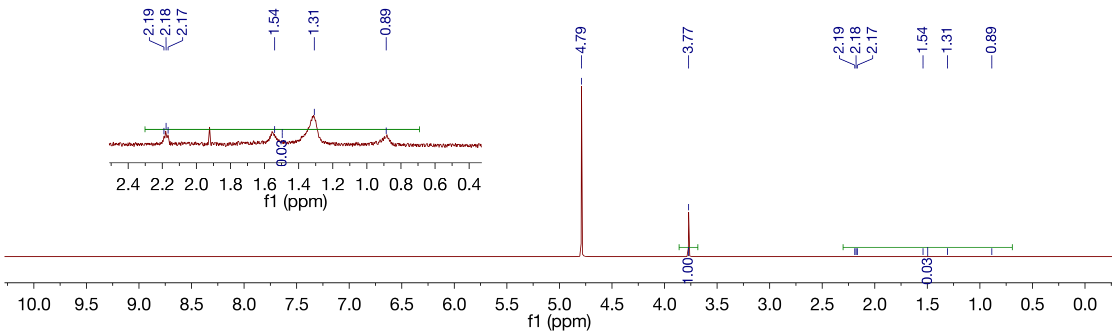

Fe(III) removal from water using the porous canola oil polysulfide (50 wt% sulfur)

A 20 mL aliquot of a 50 mg/L $FeCl_3$ solution was added to 6 centrifuge tubes (50 mL tube). The pH of this solution was 2.80. Three samples served as control experiments in which no porous polymer was added. To the remaining samples, 2.00 g of the porous polysulfide (50 wt% sulfur) was added. The polymer particle size was 1.0-2.5 mm. All samples were then placed on a rotating mixer (25 RPM) at 23 °C for 24 hours. After this time, the concentration of Fe(III) in solution was measured by taking a 1 mL aliquot and removing any solids using a benchtop centrifuge. The absorbance at 306 nm was then recorded for the sample. The results are plotted below, indicating that the porous polysulfide reduced the iron concentration to less than 2 mg/L. The pH of the water after treatment was 6.95

Figure S32: Fe(III) concentration after treating an aqueous solution of $FeCl_3$ (50 mg/mL) with the porous canola oil polysulfide (50 wt% sulfur)

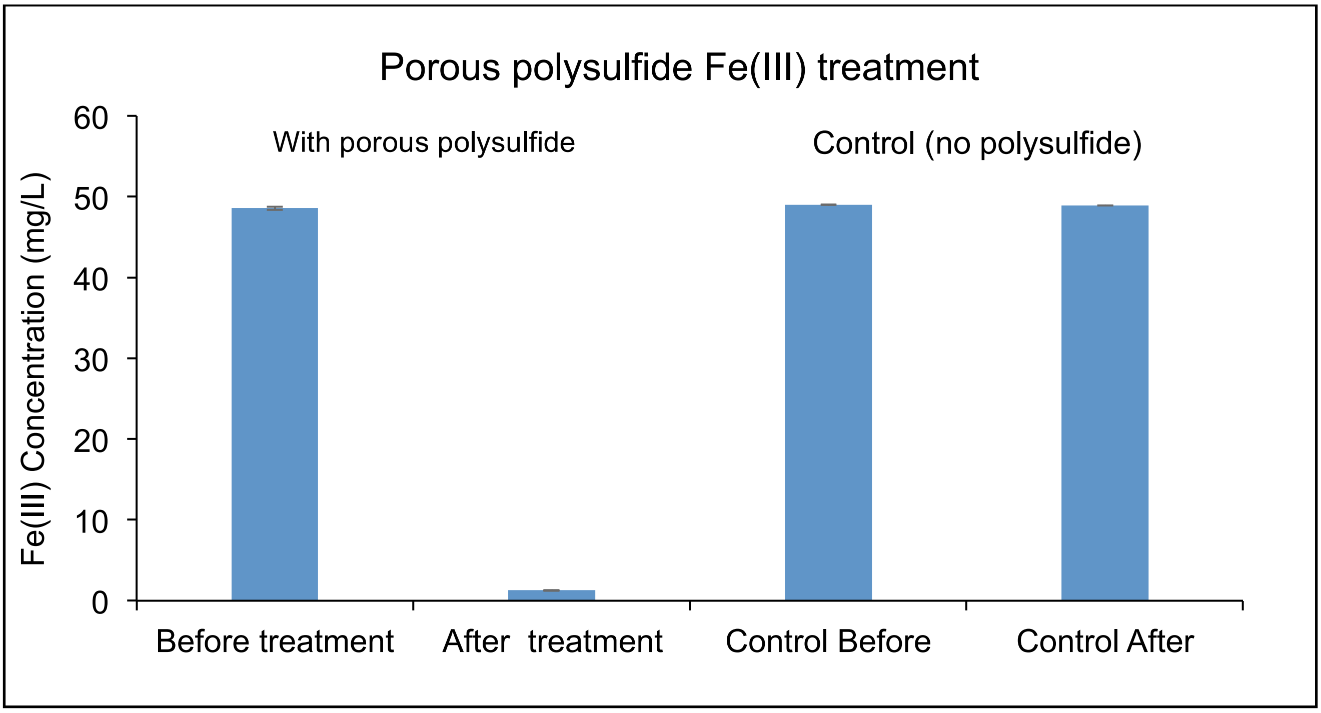

Fe(III) removal from water: porous vs non-porous polysulfide

A 20 mL aliquot of a 50 mg/L $FeCl_3$ solution was added to 12 centrifuge tubes (50 mL tube). Three samples served as control experiments in which no polysulfide was added. Into three of the tubes, 2.00 g of the porous polysulfide (50 wt% sulfur) was added. In the final three tubes, 2.00 grams of the non-porous polysulfide was added (50 wt% sulfur). All polymer particles were 1.0-2.5 mm in diameter. All samples were then placed on a rotating mixer (25 RPM) at room temperature for 24 hours. The concentration of Fe(III) was monitored for all samples by taking a 1 mL aliquot and removing any solids using a benchtop centrifuge. The absorbance at 306 nm was then recorded over a period of 24 hours. The concentration of Fe(III) in solution over time is plotted below. The porous polysulfide was able to reduce the concentration of Fe(III) to 3 mg/L within 2 hours.

Figure S33: Comparison of porous vs. non-porous polysulfide (50% sulfur) in Fe(III) removal from water

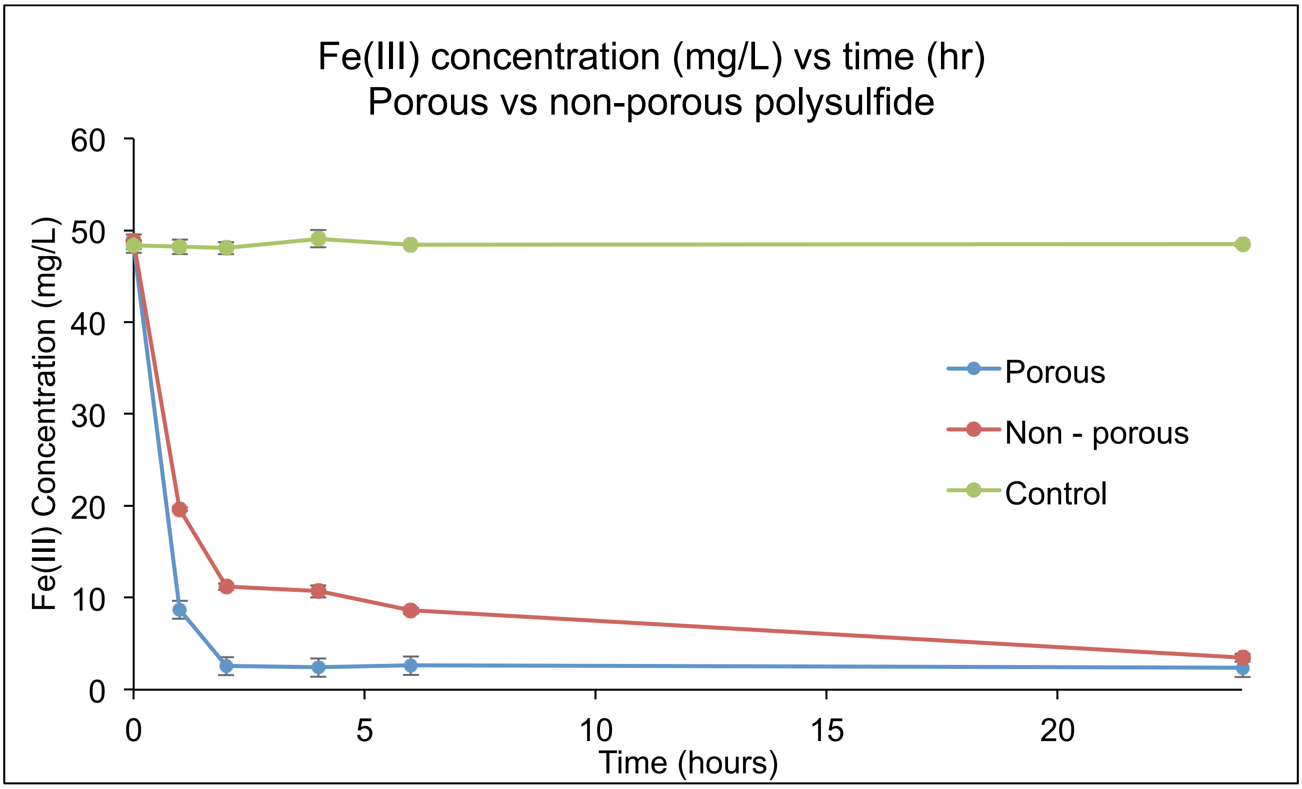

Treatment of an Fe(III) solution with different masses of the porous canola oil polysulfide (50 wt% sulfur)

The stock solution of $FeCl_3$ was prepared at 50 mg/L as described previously. This solution had a pH = 3.0, as prepared. Various masses of the porous polysulfide (50 wt% sulfur) were added to 20 mL samples of the $FeCl_3$ solution (with triplicate samples for all experiments). The polymer particles were 1.0-2.5 mm in diameter. The samples were incubated on a rotary mixer at room temperature for 24 hours, as described in the previous iron capture experiment. The absorbance was monitored at 306 nm to determine iron concentration for all samples. The results are summarised in the graph below. It was determined that 1.0 g of the porous polysulfide was sufficient for reducing the concentration of Fe(III) in a 20 mL solution of 50 mg/L Fe(III) to below 3 mg/L Fe(III).

Figure S34: Fe(III) removal from water using varying mass of the porous polysulfide (50 wt% sulfur)

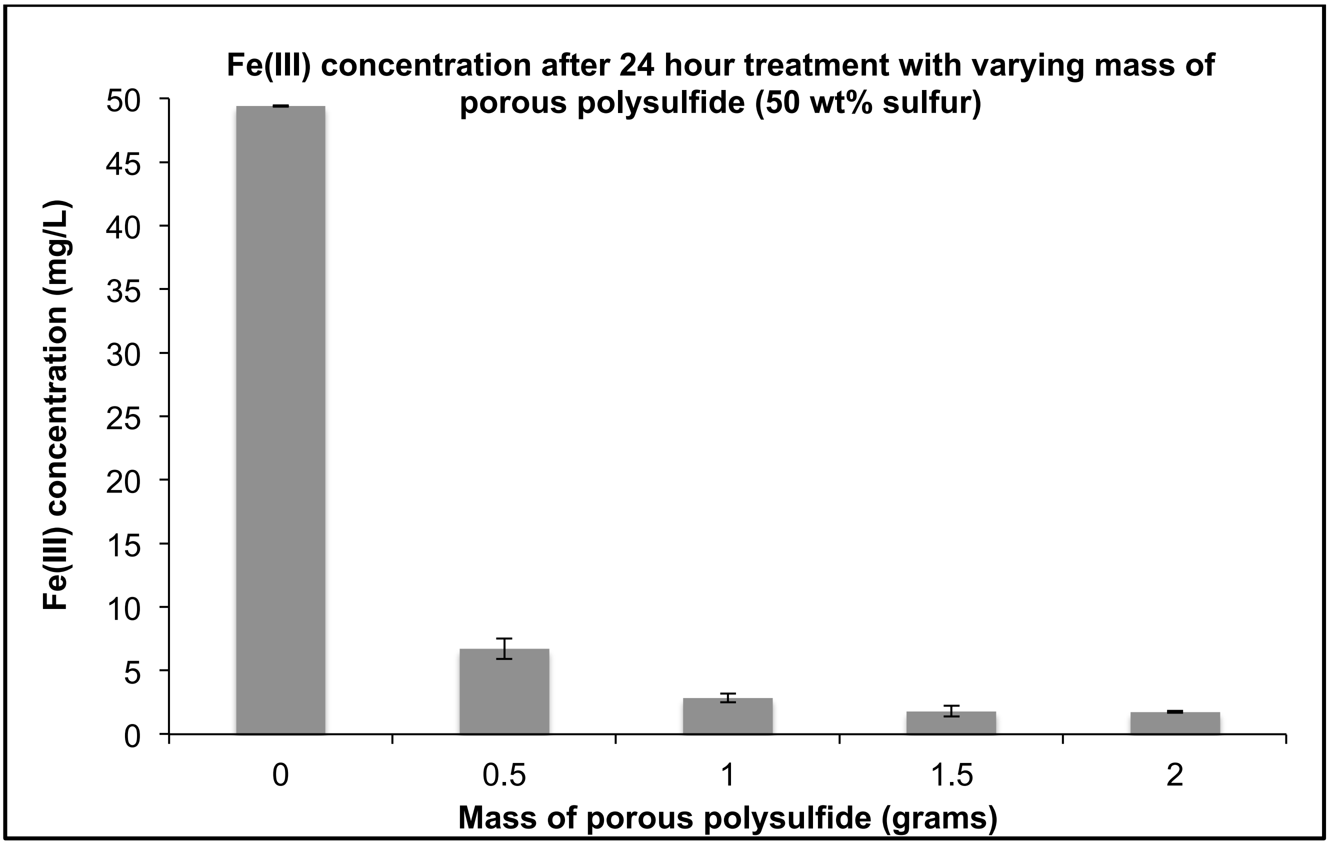

Langmuir sorption isotherm for porous polysulfide

A stock solution of $FeCl_3$ was prepared at 50 mg/L as described on page S11. This solution has a pH = 3.0, as prepared. $FeCl_3$ solutions with concentrations of 2, 5, 10, 20, 30 and 40 mg/L were then prepared by diluting the stock solution with deionised water. A 20 mL aliquot of each $FeCl_3$ solution was added to 50 ml centrifuge tubes (triplicate experiments were run for each concentration). 100 mg of the porous polysulfide was added to each solution of $FeCl_3$. All samples were then placed on a rotating mixer (25 RPM) at 23°C for 6 hours. The concentration of Fe(III) was monitored for all samples by taking a 1 mL aliquot after 6 hours of treatment and removing any solids using a bench top centrifuge and then recording the absorbance at 306 nm. The Langmuir sorption isotherm for the sorption of Fe(III) by the polysulfide sorbent was then plotted. The linearised isotherm, sorption capacity and Langmuir constants are shown below.

Langmuir Isotherm Model $$Y = \frac{Y_{max} K_L C_{eq}}{1 + K_L C_{eq}}$$

Linear form of Langmuir Isotherm model $$\frac{C_{eq}}{Y} = \frac{1}{Y_{max} K_L} + \frac{C_{eq}}{Y_{max}}$$

Where C_0 & C_{eq} = Initial and equilibrium Fe(III) concentration (mg/L)

Y = Fe(III) absorbed per gram of absorbent (mg/g) = $V(C_0 - C_e)/m$

Y_{max} = Maximum Fe(III) sorption capacity (mg/g)

K_L = Langmuir sorption constant (L/mg)

V = $FeCl_3$ solution volume (L)

M = Mass of polysulfide absorbent (g)

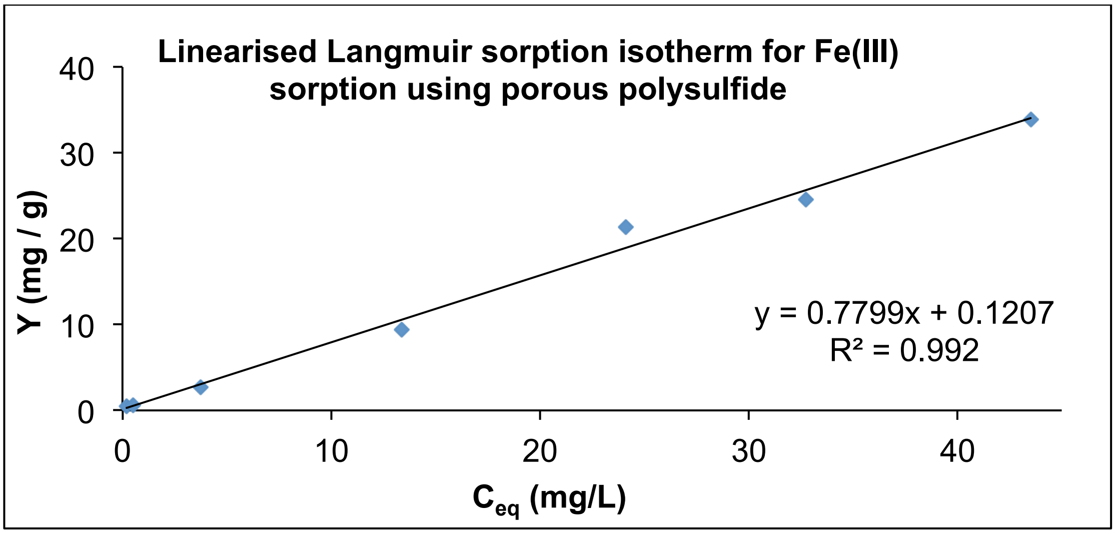

Figure S35 - Isotherm for the sorption of Fe(III) on the porous canola oil polysulfide

Y_{max} Sorption Capacity (mg/g)	K_L (L/mg)
**0.78**	**0.12**

Effect of pH on the efficiency of Fe(III) removal using the porous canola oil polysulfide

A stock solution of $FeCl_3$ was prepared at 50 mg/L as described on page S11. This solution has a pH = 3.0, as prepared. $FeCl_3$ solutions were then adjusted to pH 1, 7 and 10 by the addition of 3.0M HCl or 3.0M NaOH until the desired pH was indicated by a pH meter. 20 mL of each solution was then transferred into 50 mL centrifuge tubes along with 2 grams of the porous polysulfide. Control experiments in which no polymer was added were also carried out. All samples were then placed on a rotating mixer (25 RPM) at 23°C for 6 hours. After this time a 1 mL aliquot was transferred into a centrifuge tube. Any remaining solids were then removed using a bench top centrifuge. The concentrations of these samples were monitored by recording the UV/Vis absorbance at 306 nm. The results are shown below. Error bars indicate +/- 1 standard deviation from the mean value for the triplicate experiments.

It is clear that the polymer can remove iron from water at pH 1, reducing the iron concentration for 50 mg/L to 2.4 mg/L. At pH 7 and 10, however, the iron precipitates and can be removed from water without the addition of the polymer. To assess the ability of the polymer to bind iron, subsequent experiments were carried out at pH 3.0 so that any iron removal is attributed to the polymer and not merely precipitation. It should also be noted that when iron precipitation does occur, the polymer is still useful as it serves as a filtration media and prevents caking.

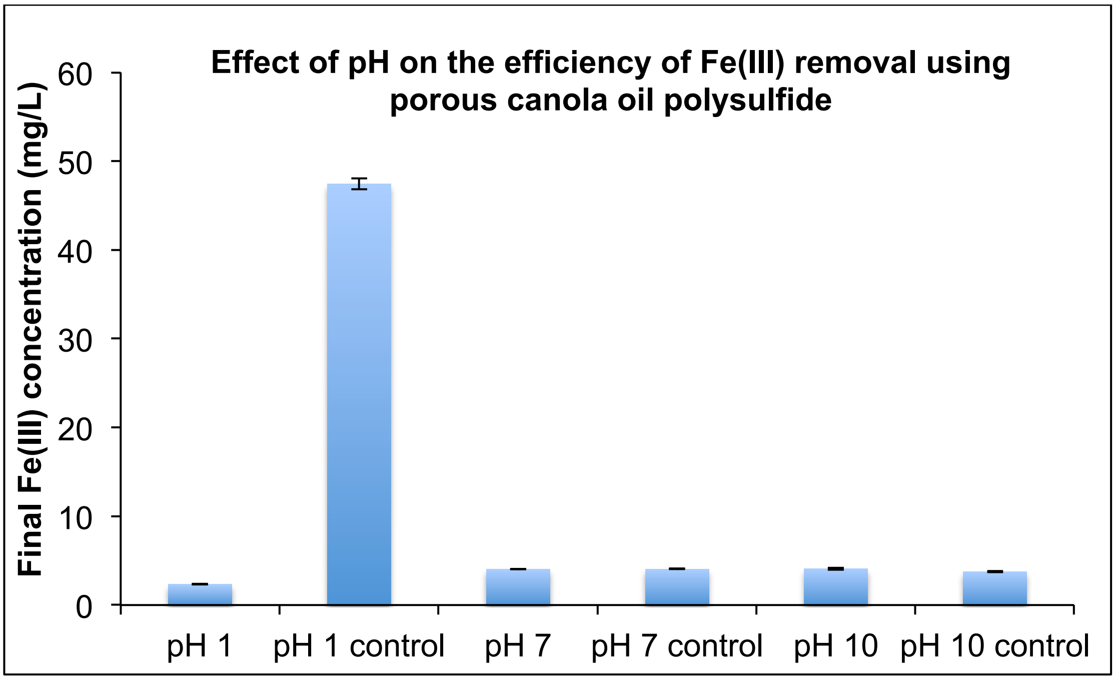

Figure S36 - Effect of pH on the removal of Fe(III) from water. Control experiments contain no polymer. The Fe(III) precipitates at pH 7 and pH 10. The polymer is required to remove the iron at pH 1.

The effect of different ions on the removal of Fe(III) from water

Initial test on solubility of $FeCl_3$ in presence of competing ions: A 20 mL aliquot of an $FeCl_3$ solution (50 mg/L, pH 3.0) was added to each of 5 glass vials. One of the following 'contaminant' salts was then added to the iron solution such that their concentration was 10 mg/L: NaCl, $CaCl_2$, KCl, Na_2SO_4. The vials were incubated at 23 ºC for 16 hours. The solution containing Na_2SO_4 led to Fe(III) precipitation so it was not considered further.

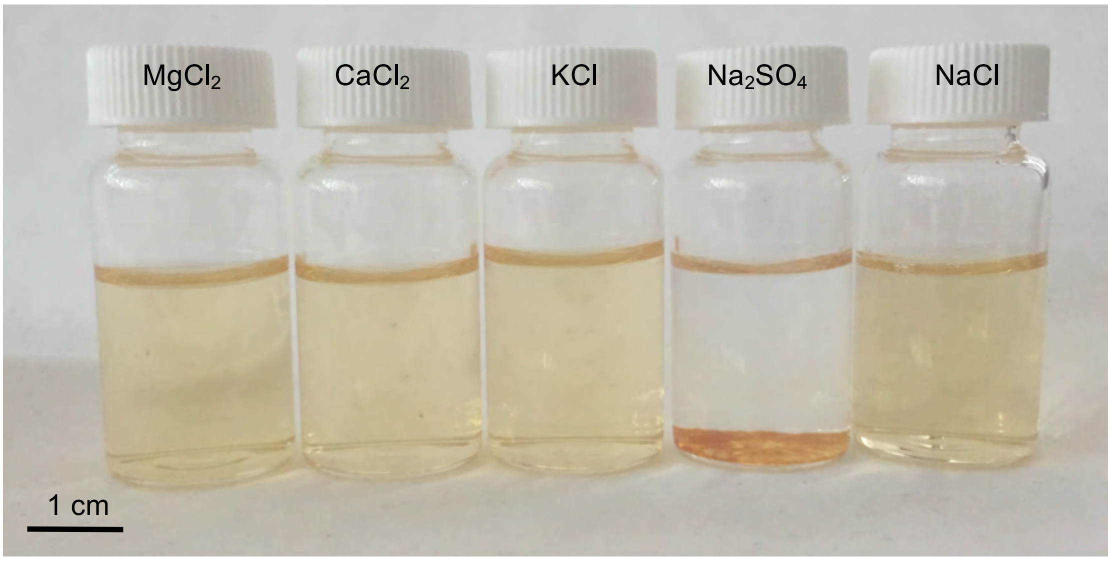

Figure S37 - Image of 50 mg/L $FeCl_3$ solutions containing 10 mg/L of $MgCl_2$, $CaCl_2$, KCl, $NaSO_4$, or NaCl.

Fe(III) sorption using the porous polysulfide in the presence of competing ions: A solution of $FeCl_3$ was prepared at 50 mg/L and then NaCl, $MgCl_2$, $CaCl_2$, and KCl were all added such that the final concentration of the 'contaminant' salts was 10 mg/L. No iron precipitated from this solution. 20 mL of this solution was transferred into 6 x 50 mL centrifuge tubes. 2 grams of porous polysulfide was added to three of the solutions, with the remaining three solutions acted as controls in which no polymer was added. All samples were then placed on a rotating mixer (25 RPM) at 23°C for 6 hours. After this time, a 1 mL aliquot was transferred into a centrifuge tube. Any remaining solids were then removed using a bench top centrifuge. The concentrations of these samples were then monitored by recording the UV/Vis absorbance at 306 nm. The results are shown in Figure S38. Error bars indicate +/- 1 standard deviation from the mean value for the triplicate experiments.

From these results it is clear that the porous polysulfide can remove Fe(III) from water (reducing the concentration of Fe(III) to 4.5 mg/L, even in the presence of the contaminant cations Na^+, K^+, Mg^{2+}, or Ca^{2+}. The increased chloride ion concentration also does not appear to make a difference in the ability of the polysulfide to capture Fe(III).

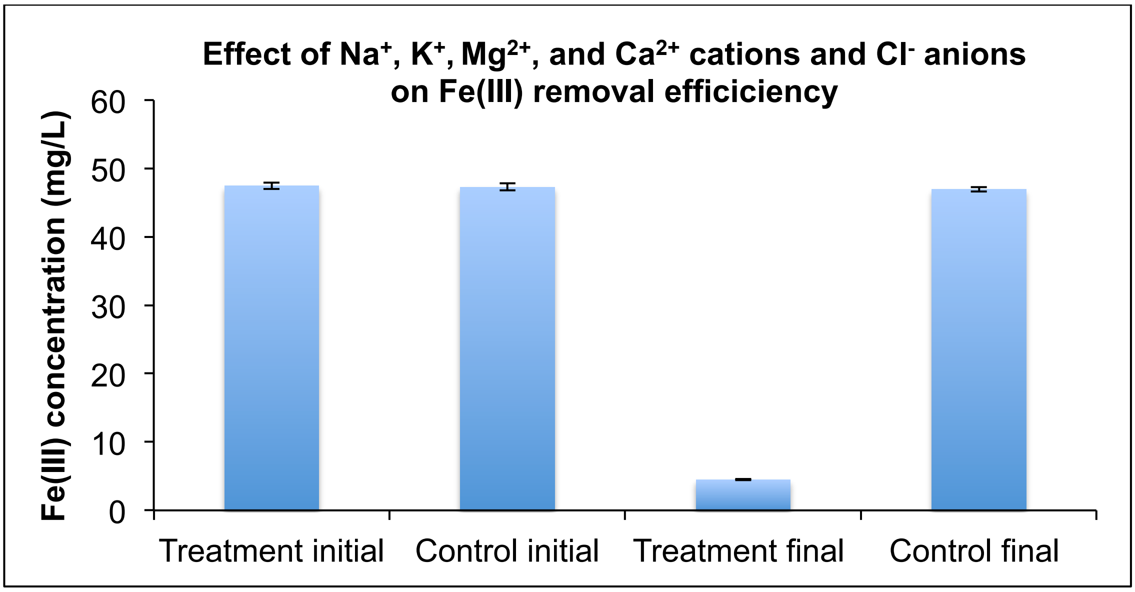

Figure S38 - NaCl, $MgCl_2$, $CaCl_2$, and KCl were all added to a 50 mg/L solution of Fe(III) chloride at pH 3.0. After 6 hours of treatment with the porous polysulfide, the iron was reduced to 4.5 mg/L Fe(III)

Kinetic analysis of Fe(III) sorption onto the porous polysulfide

A stock solution of $FeCl_3$ was prepared at 50 mg/L as described on page S11. 20 mL of this solution was transferred into 12 x 50 mL centrifuge tubes. A pH meter was used to determine the initial pH of the solution (pH = 2.8). 2, 1 and 0.5 grams of porous polysulfide were added to three solutions each, while the remaining three solutions acted as controls to which no polymer was added. All samples were then placed on a rotating mixer (25 RPM) at 23°C for 6 hours. Every hour a 1 mL aliquot was transferred into a centrifuge tube and any remaining solids were then removed using a bench top centrifuge. The concentrations of these samples were then monitored by recording the UV/Vis absorbance at 306 nm. At then end of the treatment, the pH of the water after the Fe(III) was removed was measured to be 6.95. The results of iron removal over time are shown in Figure S39. With excess polysulfide, the pseudo-first order rate constant for sorption was calculated using the 2.0 g polysulfide data.

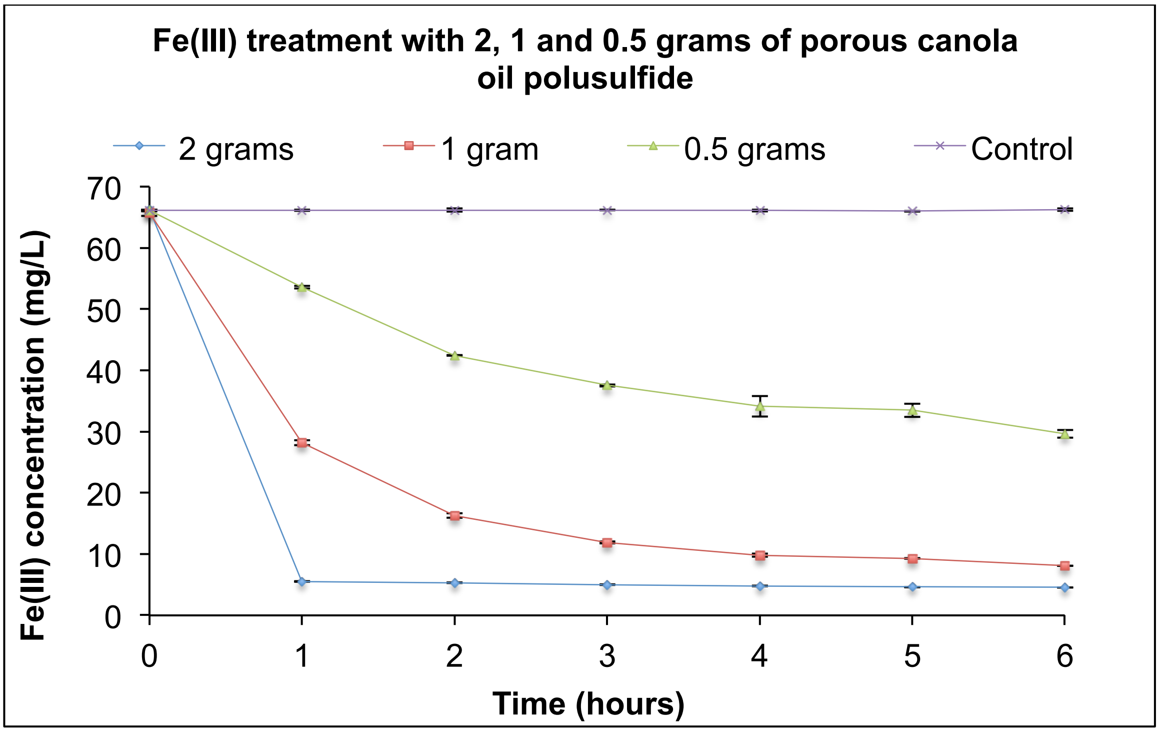

Figure S39 - Fe(III) treatment with 2.0, 1.0 and 0.5 grams of polysulfide

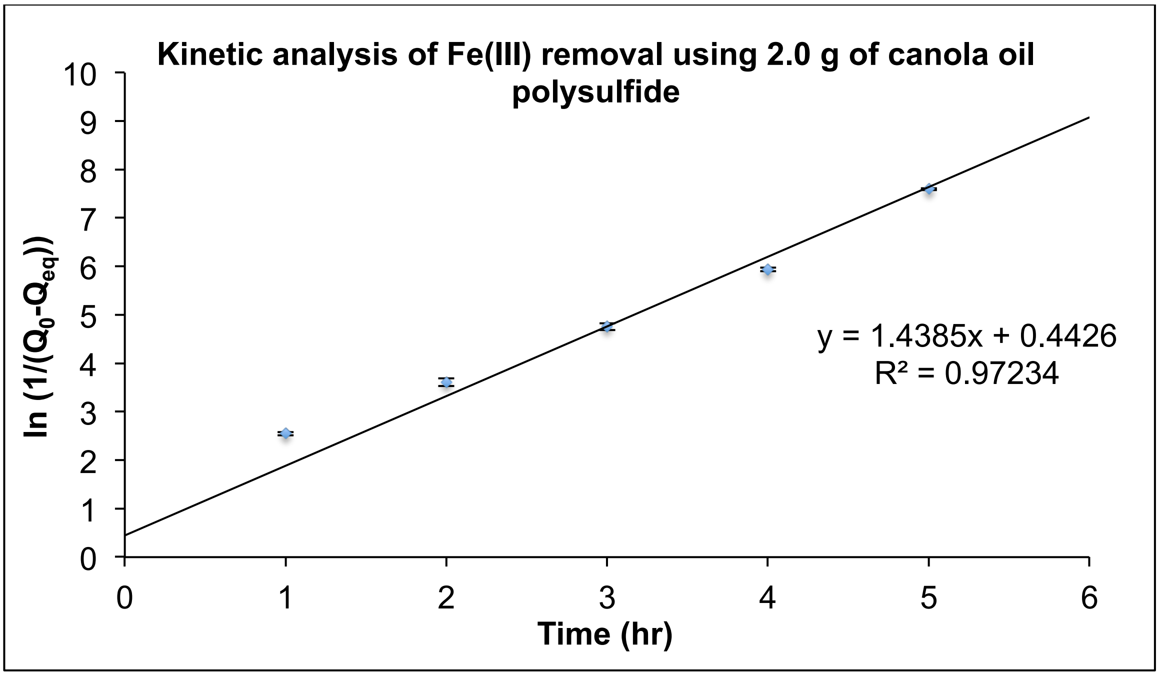

Figure S40 – Kinetic analysis for 2 grams porous canola oil polysulfide treatment of a 20 mL, 50mg/L $FeCl_3$ solution over 5 hours.

Pseudo-first order reaction: $\ln\left(\frac{1}{Q_0 - Q}\right) = K_{ad}\mathrm{t}$

Where Q_0 = Amount of iron bound at t = 0 hrs.

Q = Amount of iron bound at time = t hrs.

K_{ad} = Sorption rate constant (mg L^{-1} h^{-1})

t = time (hours)

K_{ad} = sorption rate constant = 1.44 mg L^{-1} h^{-1}

Re-use of the porous polysulfide (50 wt% sulfur) in Fe(III) removal from water

A stock solution of $FeCl_3$ was prepared at 50 mg/L as described previously. 1.50 grams of the porous polysulfide (50% sulfur) was added into a tube containing 20 mL of the stock $FeCl_3$ solution. Another tube containing only Fe(III) chloride and no polymer was used as a control. The polymer particles were 1.0-2.5 mm in diameter. The samples were incubated on a rotary mixer at room temperature for 2 hours. After 2 hours, 1 mL was transferred into a centrifuge tube and centrifuged for 2 minutes to remove any suspended solids. The absorbance was then monitored at 306 nm to determine the Fe(III) concentration. The polymer was then removed from the tube via vacuum filtration and dried in air overnight. The same polymer was then transferred to a fresh sample of $FeCl_3$ at 50 mg/L Fe(III) to see if it could still remove iron. This re-use was repeated 10 times and the concentration of iron in solution was measured after each re-use. From this data it appears that after the second use, the polymer is no longer effective at removing Fe(III) from water. The total amount of Fe(III) bound to the 1.5 grams of polysulfide after 10 treatments was 2.4 mg.

Figure S41: Re-use study of porous polysulfide in its ability to remove Fe(III) from water

Treatment Number	Concentration Fe(III) in solution after treatment (mg/L)	Fe(III) % removed
1	3	95
2	18	62
3	36	26
4	37	22
5	40	17
6	46	5
7	46	5
8	46	5
9	47	2
10	47	2

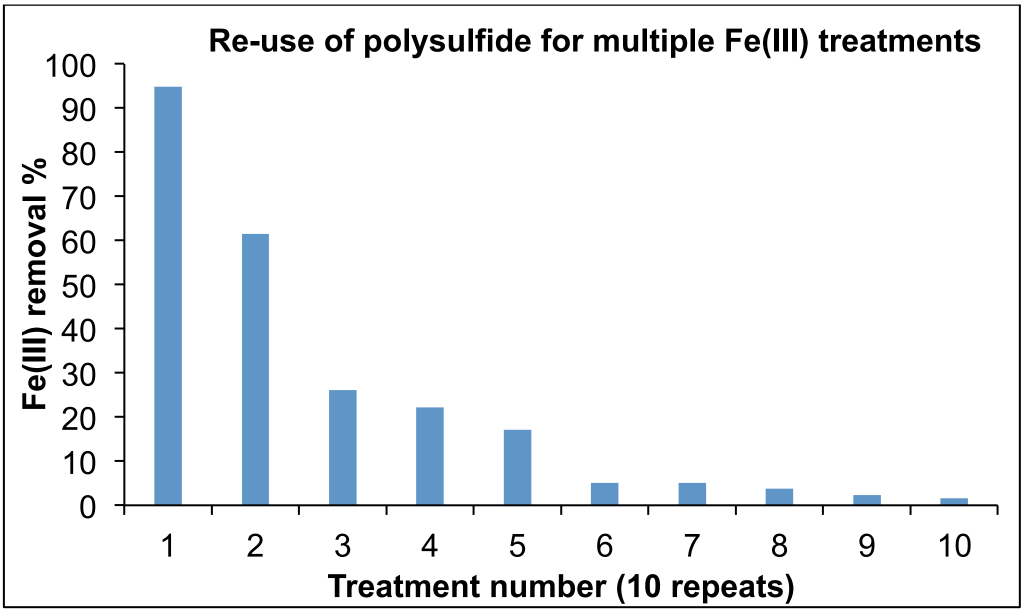

Synthesis of the porous polysulfide using recovered cooking oil (50% sulfur by mass)

NaCl (70.00 grams) was ground into a fine powder using a mortar and pestle. Waste cooking oil (used, unsaturated cooking oil from fryer, 15.00 g) was added into a 500 mL reaction vessel, which was then heated to 170 °C. Sulfur (technical grade, 15.00 g) was added to the reaction pot over a period of 5 minutes. The reaction mixture was stirred at a rate that ensured efficient mixing of the two phases. The ground up NaCl was then added over 10 minutes, and the stirring rate was continually adjusted to ensure efficient mixing. Heating was continued for an additional 10 minutes at 180 °C, over which time the reaction mixture formed a brown solid. The reaction was cooled to room temperature and removed from the reactor. The product (100.0 g) was milled for 1 minute in a blender (8.0 cm rotating blade) to give various particle sizes (typically between 0.1 mm and 3.0 cm). The particles were then transferred to a beaker and washed with 1 L of water for 4 hours with stirring. The particles were then isolated via vacuum filtration and dried in a desiccator under vacuum overnight. This washing process was repeated two more times to ensure all NaCl had been removed from the polymer. The porous polymer was a soft rubber, typically obtained in near quantitative yields (> 98%).

Figure S42: IR spectra of polysulfides made from waste cooking oil and unused food grade cooking oil (predominately canola oil in both cases)

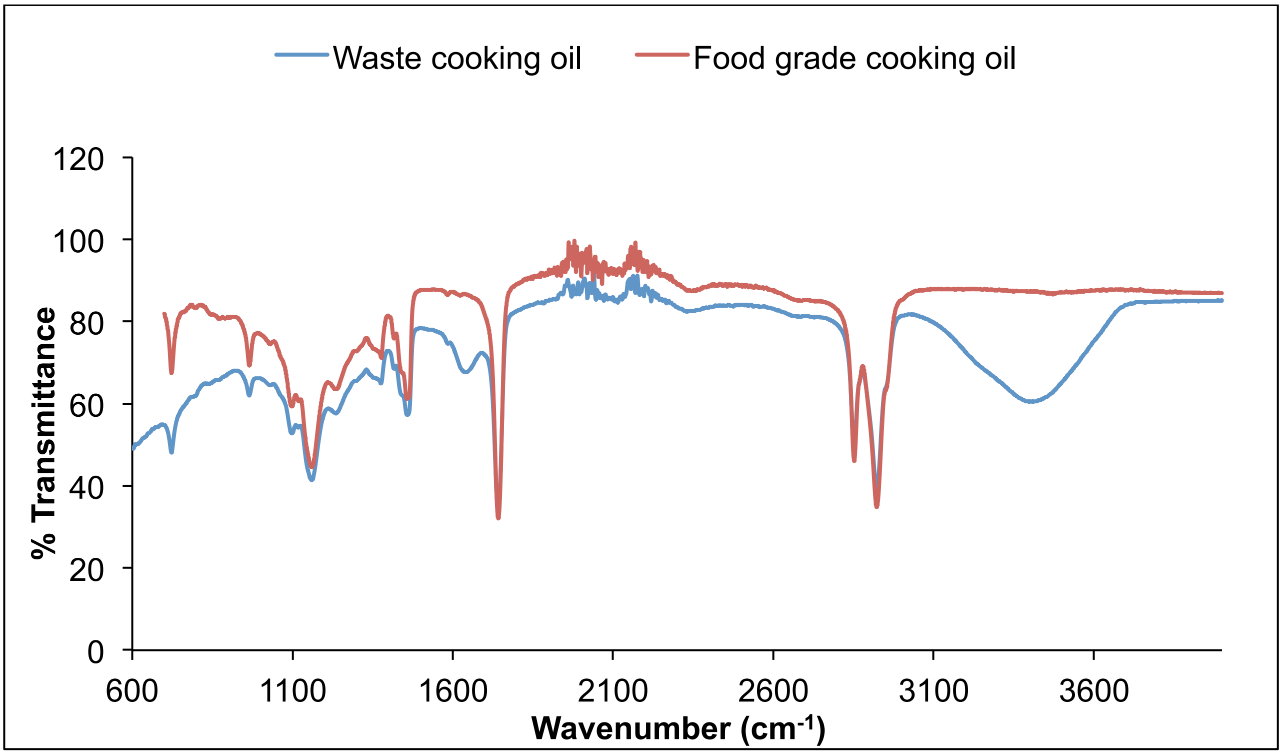

SEM analysis of the porous polysulfide prepared using waste cooking oil

Figure S43: Representative SEM of polysulfides made from waste cooking oil (50% sulfur and 50% used cooking oil by mass). A cross-section is shown to illustrate the pores and pits formed in the synthesis.

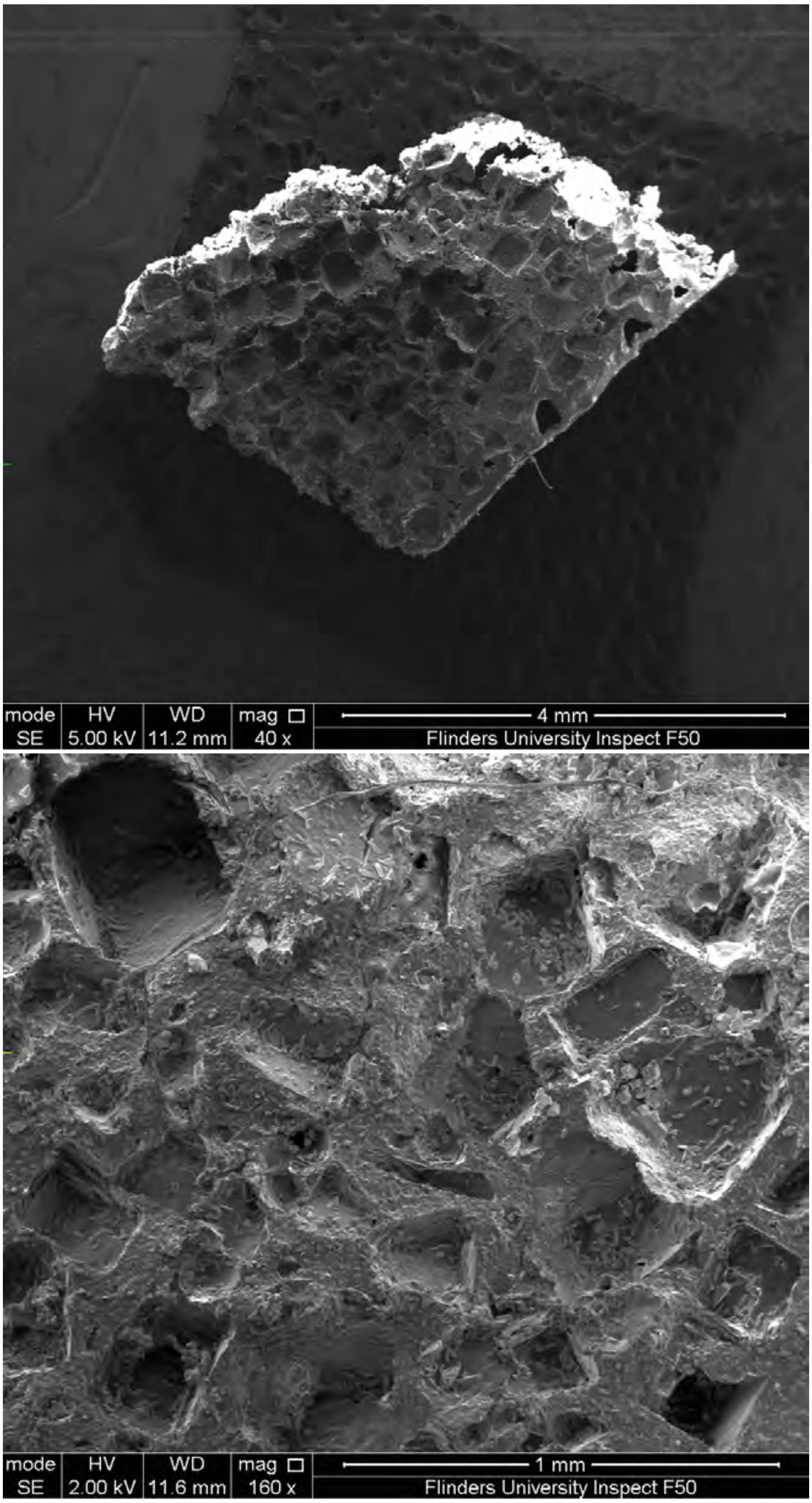

Figure S44: STA analysis of polymer prepared using waste cooking oil

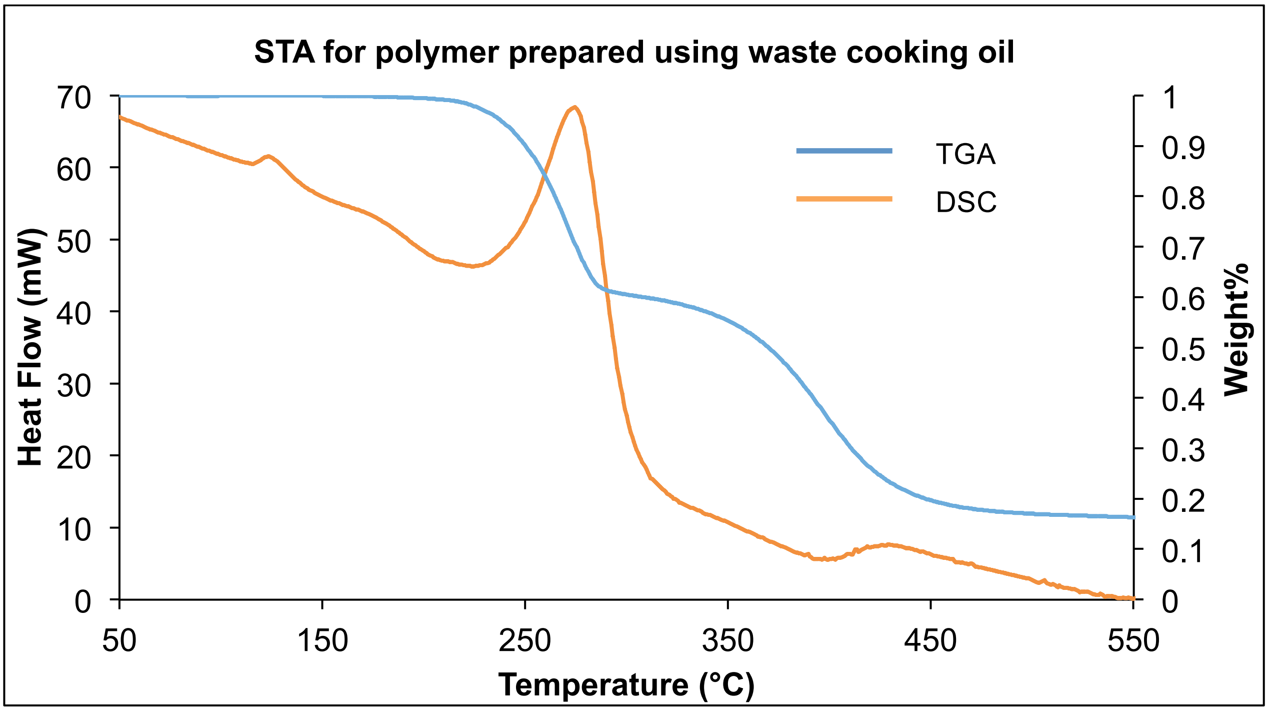

Fe(III) removal from water using a porous polysulfide prepared from recovered cooking oil

A 20 mL portion of 50 mg/L $FeCl_3$ solution was added to 6 centrifuge tubes (50 mL tube). Three samples served as control experiments in which no polymer was added. To the remaining samples, 2.00 g of the porous polysulfide (50 wt% sulfur, made from used cooking oil) was added. All samples were then placed on a rotating mixer (25 RPM) at room temperature for 24 hours. After this time, the concentration of Fe(III) in solution was measured by taking a 1 mL aliquot and removing any solids using a benchtop centrifuge. The absorbance at 306 nm was then recorded for all samples.

Sample	Average Fe(III) Concentration (mg/L)
No Polymer	48.6 ± 0.2
2h treatment with polymer	2.0 ± 0.2

Figure S45: The porous polysulfide prepared from waste cooking oil and sulfur was effective at removing Fe(III) from water.

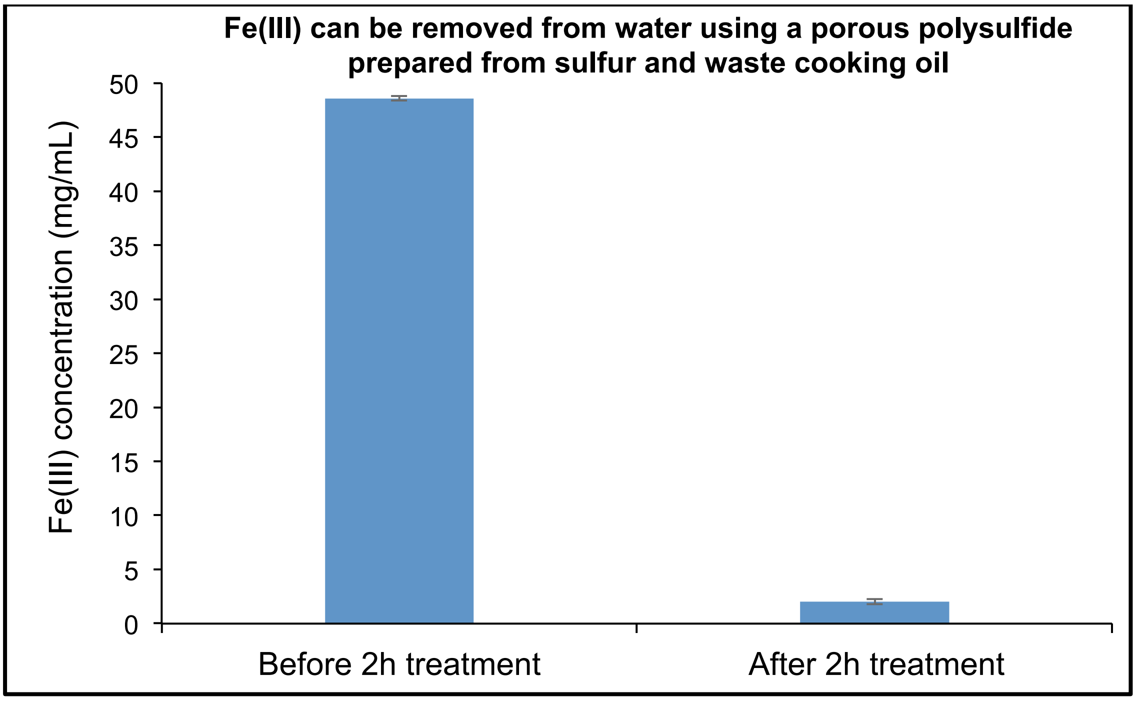

Porous canola oil polysulfide synthesis using a microwave reactor (50% sulfur, 6 gram scale)

3.00 grams of elemental sulfur was added to a 500 mL RBF equipped with an oval-shaped stirring bar (12 × 15 mm). 3.00 grams of canola oil was then added to the flask followed by the addition of 14.0 grams of sodium chloride. The flask was placed into a Startsynth front-loading microwave reactor on top of a Weflon flask holder with a condenser attached to the flask. The stirred mixture was then irradiated with microwaves. The power of the microwave was set 1200 W and the reaction was heated at constant power for approximately 8 minutes and 50 seconds. The heating profile is shown below. The mixture was stirred at 10% of the maximum speed until the sulfur had melted. At this point the stirring rate was increased to 70% of the maximum speed and maintained at this level for the remainder of the synthesis. After 8 minutes and 50 seconds had passed the irradiation was stopped, but stirring was continued until the cross-linking prevented further stirring (approximately 1 minute after irradiation ceased). The flask was then lifted off of the Weflon stand to cool the reaction vessel. The product was then removed from the flask using a metal spatula.

The product was then transferred into a 500 mL beaker with 250 mL of DI H_2O and stirred for 24 hours to remove sodium chloride from the polymer. After 24 hours the product was filtered by vacuum filtration and rinsed with 200 mL of distilled water. The product was then re-washed by stirring in 200 mL DI water for 1 hour. The product was then isolated by vacuum filtration and dried in air over night. Near quantitative yields were obtained (>98%).

Notes: For 6 gram scale synthesis a 500 mL round bottom flask must be used so that the reaction mixture is evenly distributed around the bottom of the flask and heated evenly. The stirring rate must be monitored throughout the reaction because of the changes in viscosity. It is critical to ensure efficient mixing of the sulfur and canola oil. If the mixture isn't stirred efficiently, the reaction can phase separate and gel in a non-uniform manner.

Figure S46: Heating and power profile for microwave-based synthesis of porous canola oil polysulfide (50% sulfur)

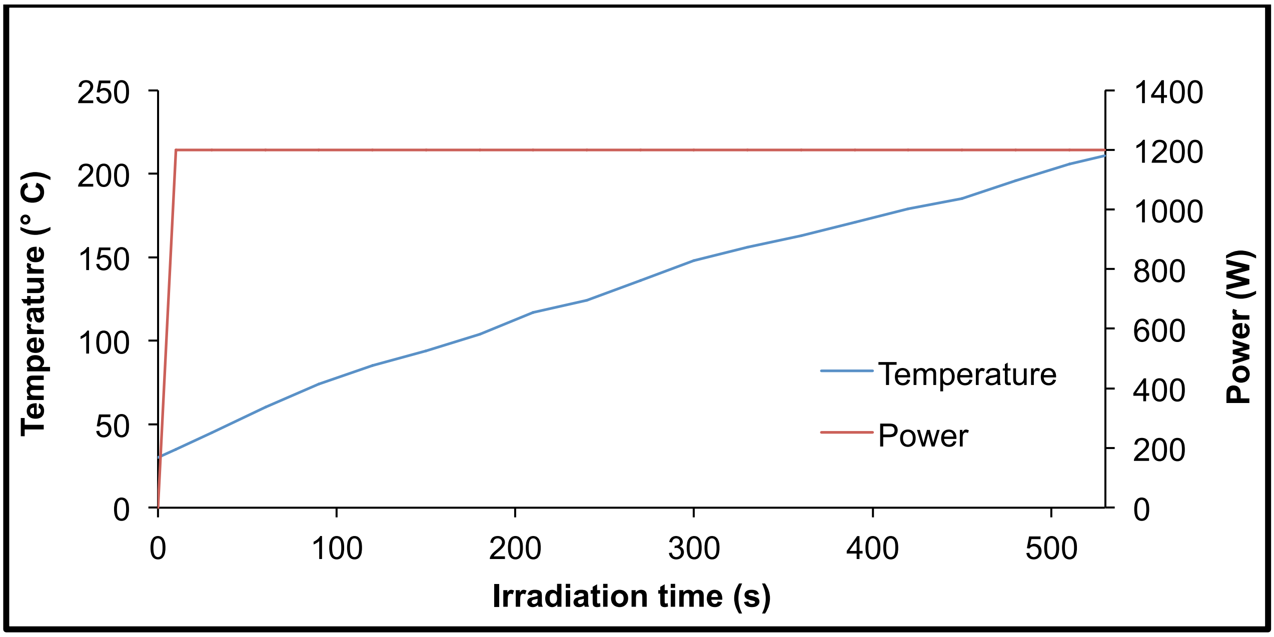

Figure S47: Microwave irradiation is effective at promoting the reaction of sulfur and canola oil in the preparation of a porous polysulfide (50 wt% sulfur). A) Canola oil. B) Canola oil and sulfur. C) Canola oil, sulfur, sodium chloride. D) Product as formed in flask from microwave irradiation. E) Isolated porous polysulfide after washing out sodium chloride.

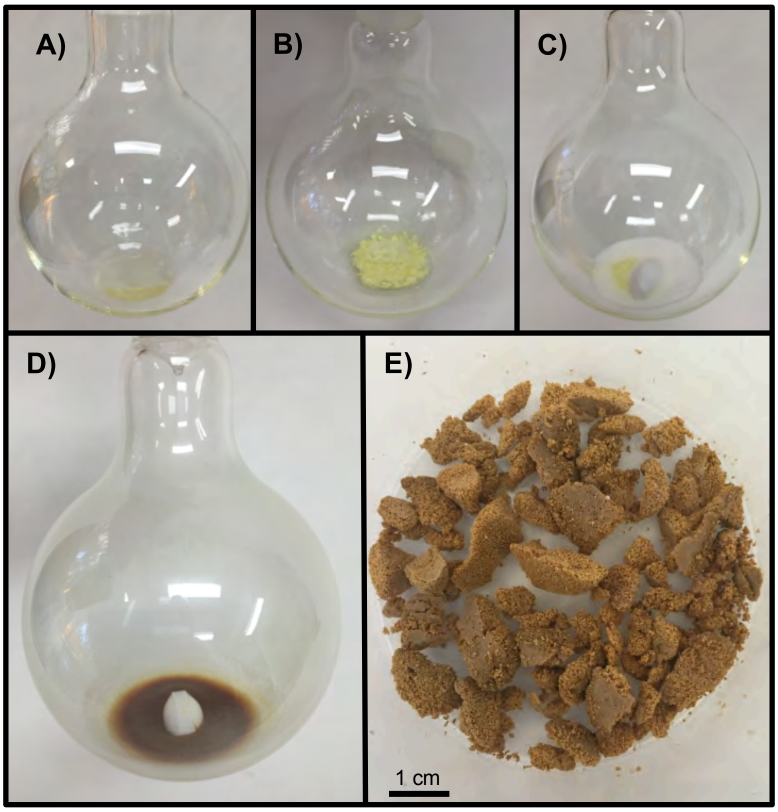

Porous canola oil polysulfide synthesis using a household microwave (50% sulfur, 30 gram scale)

15.00 grams of elemental sulfur was mixed with 15.00 grams of canola oil in a 10.5 cm diameter Pyrex dish, followed by the addition of 70.0 grams of NaCl. All components were mixed with a spatula to form a viscous paste. The mixture was then placed into a household microwave (1100W) and irradiated for 4 minutes and 30 seconds. The mixture was then removed from the microwave and mixed using a metal spatula for 30 seconds, before being returned to the microwave. The mixture was then irradiated for a further 30 seconds and then mixed with a metal spatula for 30 seconds or until the product solidified. A final yield of >98% was typically achieved using this method.

Notes: Do not let the temperature exceed 200 °C, otherwise the polymer can decompose and emit H_2S. The temperature can be monitored periodically (between irradiations) by using a non-contact hand-held IR thermometer. Limit the amount of time during the stirring steps to prevent the reaction from cooling down too much. Ensure thorough mixing of the reaction has occurred, as insufficient mixing will result in phase separation and a non-uniform gel formation. Microwaves with lower power than 1100W will require longer irradiation times for the reaction to reach completion. In this case a similar procedure is used whereby the sample is irradiated until the initial colour change is noted. After this point, 30 second periods of irradiation, followed by 30 seconds of stirring, are performed until the product is formed.

Figure S48: a) Reaction mixture before irradiation, b) Polysulfide product directly after irradiation, c) Final porous polysulfide product after washing.

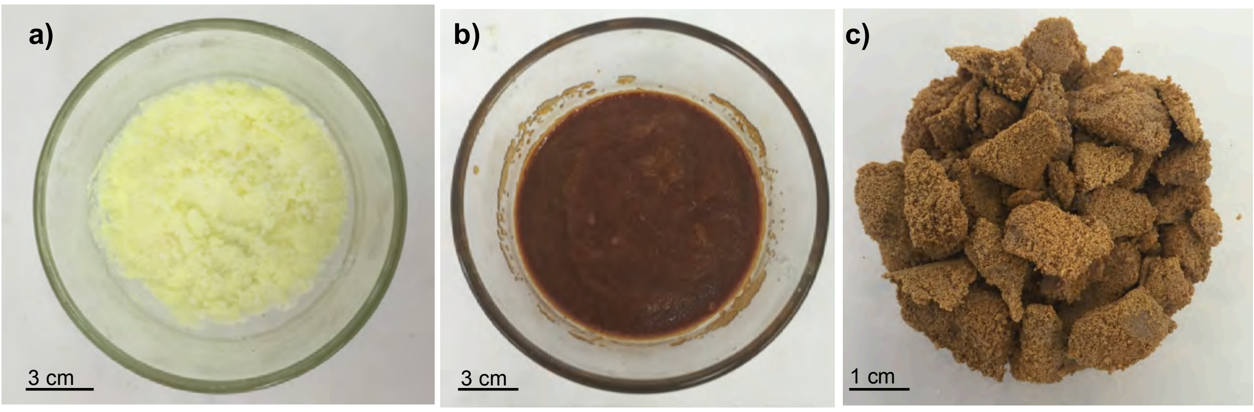

Figure S49: IR spectra of the porous canola oil polysulfide (50% sulfur) prepared by microwave irradiation

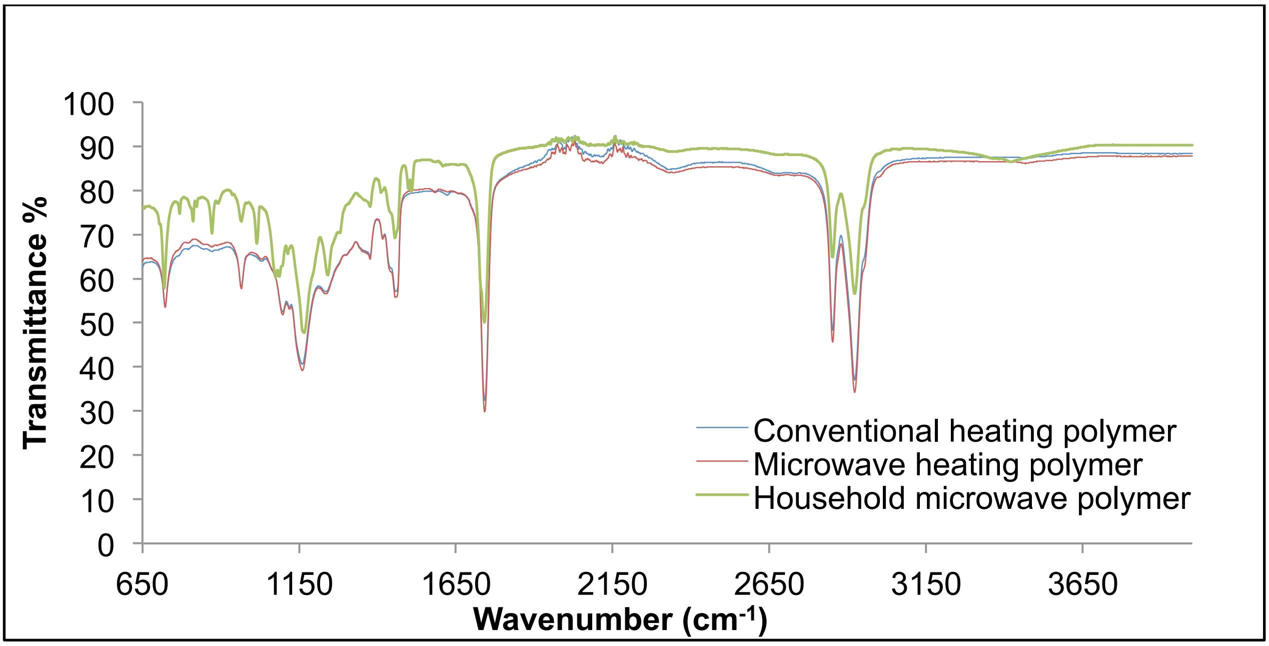

Figure S50: SEM micrograph of canola oil polysulfide (50 wt% sulfur) prepared using a laboratory microwave reactor.

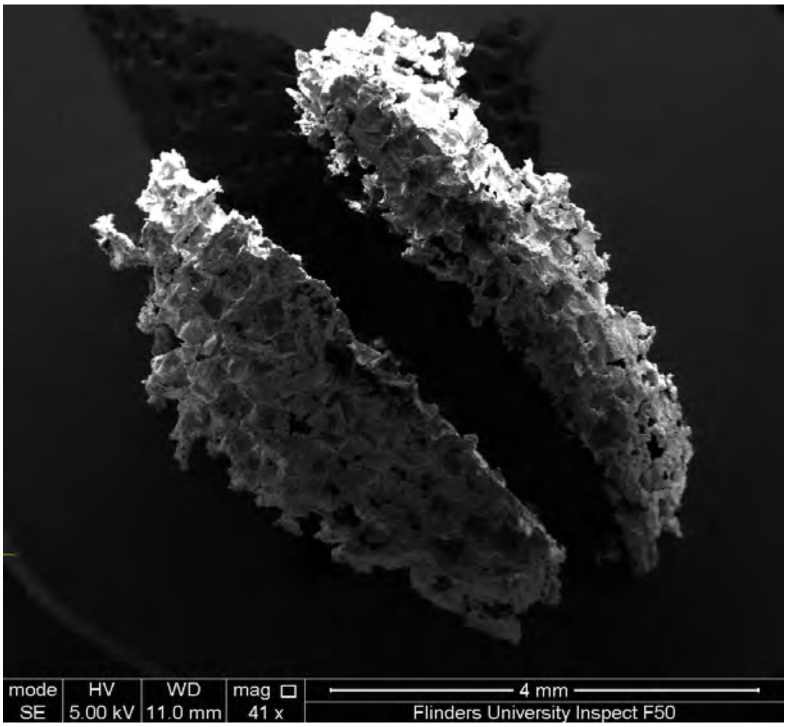

Figure S51: Representative SEM micrograph of the porous canola oil polysulfide (50% sulfur) prepared with a household microwave

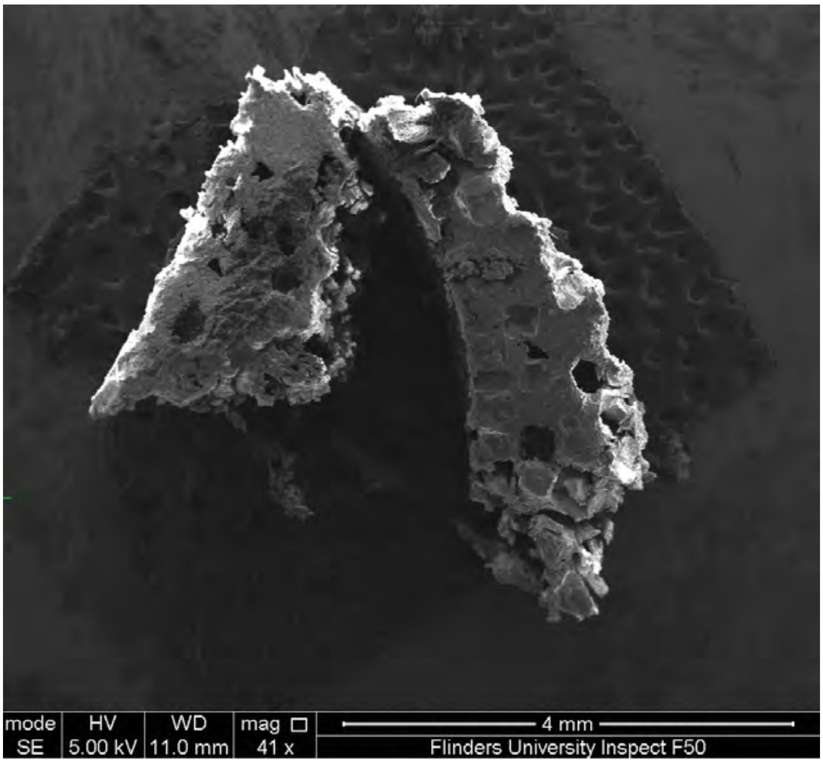

Figure S52: STA analysis of the porous canola oil polysulfide (50% sulfur) prepared with a laboratory microwave reactor

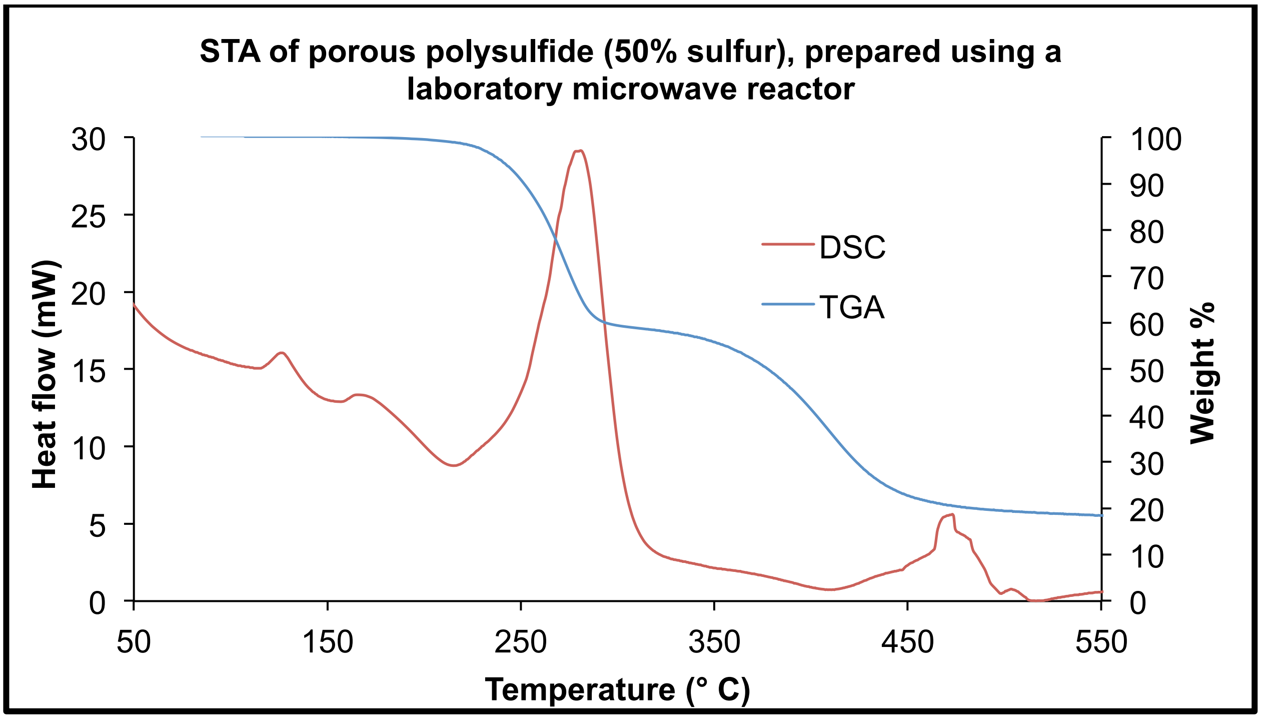

Figure S53: STA analysis of the porous canola oil polysulfide (50% sulfur) prepared with a household microwave

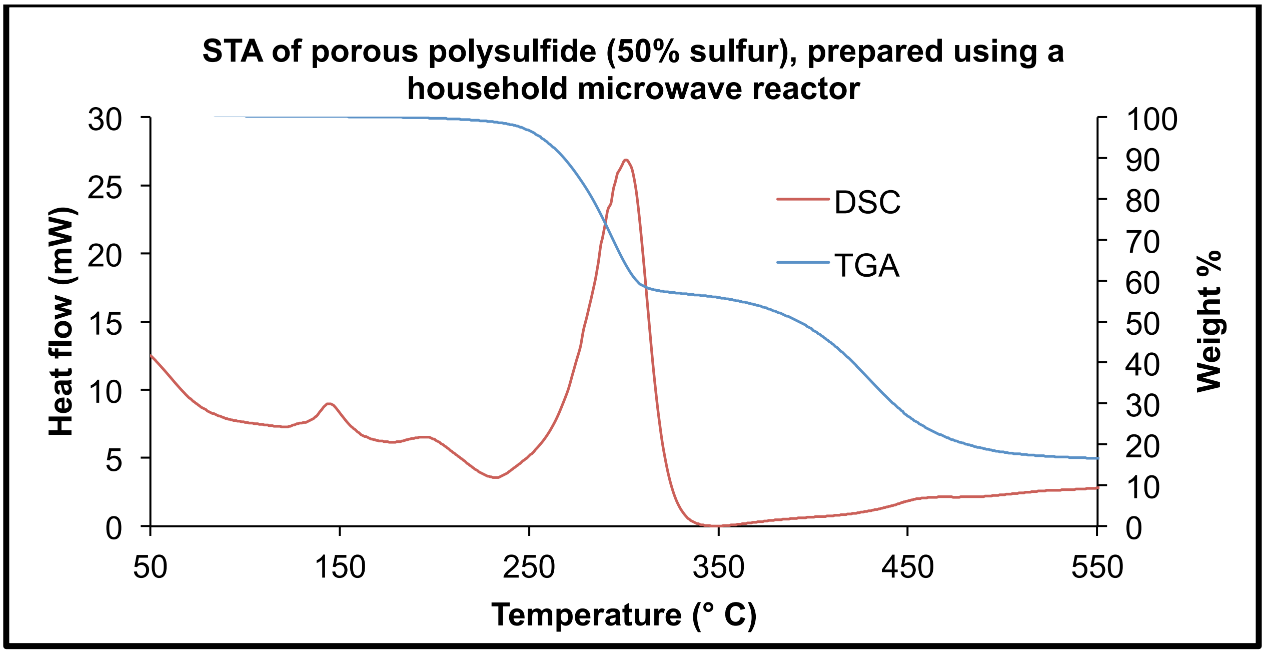

Fe(III) treatment using polysulfide prepared using microwave irradiation

A 50 mg/L solution of iron (III) chloride was prepared by adding 5 mg of $FeCl_3$ into a 100 mL volumetric flask which was then filled up to the 100 mL mark with distilled water. Nine experiments were then prepared (triplicate testing of the polymer prepared using a laboratory microwave reactor, triplicate testing of the polymer prepared using conventional heating, and triplicate controls in which no polymer was used). Accordingly, 1.0 gram of the porous canola oil polysulfide (50% sulfur), prepared using microwave irradiation, was transferred into 3 separate 50 mL centrifuge tubes. Second, 1.0 gram of the porous canola oil polysulfide (50% sulfur), prepared using conventional heating, was transferred into 3 separate 50 ml centrifuge tubes. Then, 20 ml of the $FeCl_3$ (50 mg/L) solution was transferred into each centrifuge tube containing polymer. 20 ml of the $FeCl_3$ (50 mg/L) solution was also transferred into three centrifuge tubes containing no polymer for a negative control. All nine tubes were placed in a rotary mixer and mixed at 25 rpm for 2 hours. Finally 1 mL aliquots of each sample were transferred into 1 mL centrifuge tubes and centrifuged for 1-2 minutes. These samples were transferred into cuvettes and their UV/Vis absorbance at 306 nm was recorded to determine the Fe(III) concentration. Under these conditions, the polymer prepared using a microwave removed 94% of the Fe(III) and the polymer prepared using conventional heating removed 93% of the Fe(III).

Figure S54: Fe(III) removal using a porous canola oil polysulfide prepared using microwave irradiation is as effective as the same polymer prepared using conventional heating.

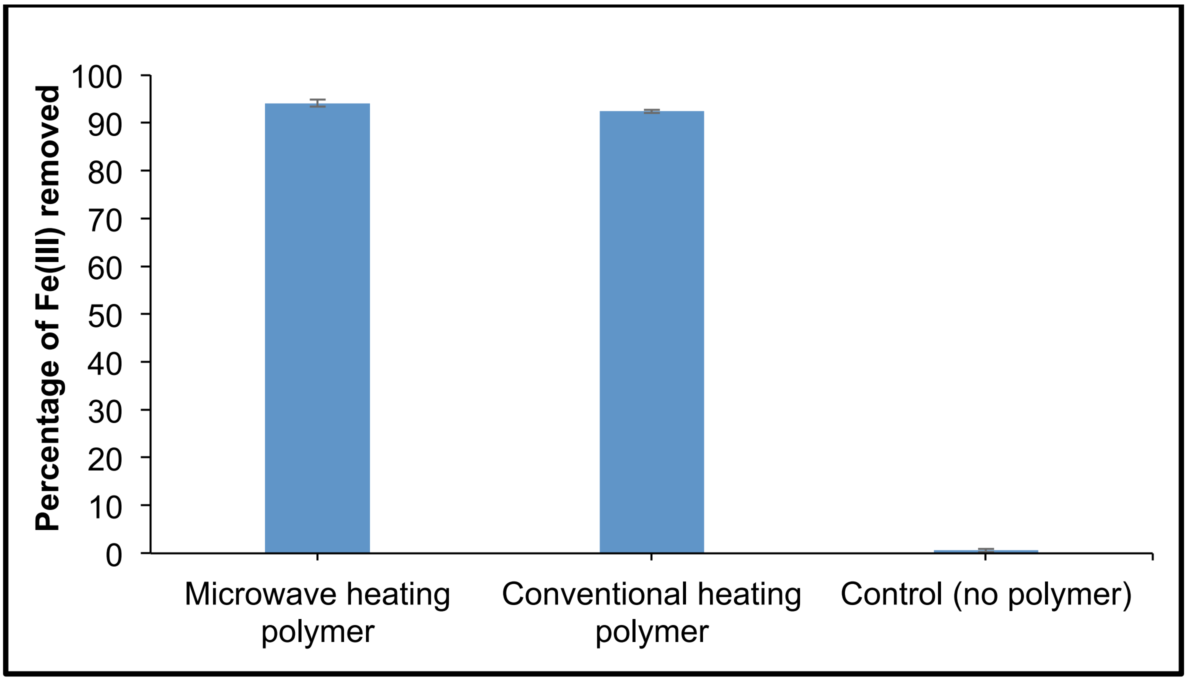

References

1. Worthington, M. J. H.; Kucera, R. L.; Abuquerque, I. S.; Gibson, C. T.; Sibley, A.; Slattery, A. D.; Campbell, J. A.; Alboaiji, S. F. K.; Muller, K. A.; Young, J.; Adamson, N.; Gascooke, J. R.; Jampaiah, D.; Sabri, Y. M.; Bhargava, S. K.; Ippolito, S. J.; Lewis, D. A.; Quinton, J. S.; Ellis, A. V.; Johs, A.; Bernardes, G. J. L.; Chalker, J. M. *Chem. Eur. J.* **2017**, *23*, 16219-16230.
